# Feeling safe: an empirical-ethical analysis of vulnerability in (es)ketamine treatment

**DOI:** 10.1186/s12910-026-01637-7

**Published:** 2026-10-08

**Authors:** Adrian Kind, Josephine Görke, Hannah Smirr, Christina Ni, Karen Yan, Dimitris Repantis

**Affiliations:** 1 Department of Psychiatry and Neurosciences, Charité – Universitätsmedizin Berlin, corporate member of Freie Universität Berlin and Humboldt-Universität zu Berlin, Campus Charité Mitte, Berlin, Germany; 2 German Center for Mental Health (DZPG), partner site Berlin-Potsdam, Berlin, Germany; 3 Department of Psychology and Education, Freie Universität Berlin, Berlin, Germany; 4 Department of Psychology, Faculty of Life Sciences, Humboldt-Universität zu Berlin, Berlin, Germany; 5 Institute of Philosophy of Mind and Cognition, National Yang Ming Chiao Tung University, Taipei, Taiwan

**Keywords:** Ketamine, Esketamine, Felt safety, Vulnerability, Empirical bioethics, Set and setting, Therapeutic alliance, Informed consent, Qualitative research

## Abstract

**Background:**

Treatments with acute consciousness-altering effects, such as (es)ketamine, place patients in a state of heightened vulnerability with reduced capacity for self-advocacy and self-regulation. Since feeling safe is a precondition for consenting to begin, and remaining in, treatment, this raises distinct ethical demands on care. Which features of the setting enable patients to feel safe, and how patients' and clinicians' understandings of the treatment relate to these requirements, remains unclear. This study identifies patient-, clinician- and setting-related factors shaping the experience of safety during (es)ketamine treatment and derives ethically grounded recommendations, distinguishing clinical safety, felt safety and ethical protection and analyzing their relations through a framework of induced, layered vulnerability.

**Methods:**

Semi-structured interviews were conducted with 14 patients who had received intranasal esketamine (Spravato®) or IV ketamine infusion treatment and 10 clinicians administering these treatments in German psychiatric settings, and analyzed using structuring qualitative content analysis complemented by type-building analysis. Pre-treatment concerns and the experience of safety were analyzed from the patient interviews; clinician interviews informed the broad coding and the comparison of patients' and clinicians' models of ketamine's mechanism of action.

**Results:**

Three types of pre-treatment concern, three overlapping modes of experiencing safety, and three models of ketamine's mechanism of action were identified, each associated with distinct treatment requirements. Therapeutic presence, active physical grounding, and structured post-acute transitional support (“landing aid”) were described by participants as central to feeling safe. Clinicians more often conceived of ketamine as a catalyst of transformation, patients as temporary relief or symptom management, indicating potential misalignment regarding preparation and integration.

**Conclusions:**

Safety in (es)ketamine treatment is relationally created and individually differentiated rather than uniform, supporting an ethical case for flexible, patient-centered care above a non-negotiable floor of clinical safety. Because patients in altered states depend acutely on those who induced that state, minor clinician behaviors and the post-acute transition carry moral weight bearing on informed consent and the duty of care, yet are modifiable with few resources. Recommendations are graded by strength; one is restricted against our own empirical finding where the vulnerability analysis requires it. As an exploratory study, the findings require confirmation in larger, more diverse samples.

**Supplementary Information:**

The online version contains supplementary material available at https://doi.org/10.1186/s12910-026-01637-7.

## Background

(Es)ketamine has become an established treatment in psychiatry. In 2019, the FDA and EMA approved esketamine nasal spray for treatment-resistant depression; intravenous (IV) ketamine infusions, meanwhile, remain a widely used off-label treatment for the same indication and for various other psychiatric disorders [1–4].

Unlike most psychopharmacological treatments for depression, (es)ketamine produces rapid psychoactive effects, reported as comparable to those of classical psychedelics [5–7]; although (es)ketamine is more often framed as “dissociative” [8, 9], it too elicits mystical-type experiences [10–12]. Intranasal esketamine and the forms of ketamine administration differ pharmacologically and in their subjective effects; we focus on their shared consciousness-altering character, which is what makes them ethically salient. Patients learn, from their own reading or from clinicians, about acute dissociative and psychedelic-like effects that are often experienced as inducing vulnerability [13], and unless they feel prepared and perceive the context as safe, the constellation conceptualized as “set and setting” [14], they may hesitate to enter or continue treatment for reasons that are not strictly biomedical. Because ketamine is often one of the last options in treatment-resistant depression, the opportunity costs of such avoidance can be substantial. Clinical institutions therefore bear a responsibility to design settings that support patients’ sense of safety, which requires knowledge of patients’ own expectations, concerns and experiences.

The consciousness-altering properties of esketamine have been formally acknowledged by both major regulatory agencies as grounds for specific monitoring requirements. The FDA’s approval of intranasal esketamine for depression in 2019 was accompanied by a Risk Evaluation and Mitigation Strategy under which patients must be monitored by a healthcare provider for at least two hours after each administration and may not take the medication home [15]. The EMA’s Summary of Product Characteristics requires a post-administration observation period in an appropriate clinical setting, under supervision until the patient is considered clinically stable and ready to leave, an open-ended formulation that places the judgment of readiness with the clinician rather than the clock [16]. Both requirements implicitly acknowledge that the transition out of a session is a period of continued vulnerability requiring structured clinical attention, and they lend institutional support to treating post-acute transitional care as a standard component of treatment rather than an optional addition.

### Relevant previous research

Understanding patients’ expectations, attitudes, and experiences is largely a task for qualitative research. Ketamine treatment has been examined through case studies (e.g. [17]), focus groups [18, 19], thematic analysis [20], and interpretative phenomenological analysis [21, 22]. Yet few studies focus specifically on “set and setting,” that is, the treatment context, interpersonal dynamics, and treatment arrangements, and most remain centered on the acute psychological effects of the substance.

Griffiths et al. [22] found, through interpretative phenomenological analysis of patients receiving ketamine infusions, that patients weigh risks and benefits before treatment, that trust and rapport shape willingness to proceed, and that feeling safe and “looked after” enhances the experience, but remained general about what concretely constitutes a helpful relationship or “safe” setting. Mollaahmetoglu et al. [20] reported limited alternatives as a central motivator, appreciation of a “professional” and “controlled” setting, and the influence of prior drug experience or a first IV ketamine infusion on expectations, a point echoed by Stockwell et al. [13].

Walaszek et al.‘s narrative review [23] confirmed the centrality of set and setting in determining whether acute effects were experienced as therapeutic or distressing and the motivational role of desperation after repeated treatment failures: preparation, sustained presence and debriefing enabled patients to tolerate dissociation and anxiety, whereas isolation, poor information or inaccessible staff intensified fear and disengagement, consistent with Breeksema et al.‘s finding that distress is common across all treatment stages [10]. The review concluded that ketamine should be embedded in ongoing relational care rather than treated as an isolated pharmacological procedure.

Much of this depth derives from Breeksema et al.‘s phenomenological analyses [10, 21], which show that the capacity to “let go” during overwhelming states is shaped by clinician presence and emotional support, though their focus remains on the phenomenology of the experience rather than on specific clinician behaviors. Stockwell et al. [13] reported that therapeutic intention, mindfulness, therapist experience, music and “spiritual cues” supported the experience and that vulnerability extended into aftercare, but these findings, too, remain at a high level of generality.

Across this literature, the importance of preparedness, trust, and a supportive setting is well established, yet what concretely makes patients feel safe, which clinician behaviors stabilize or destabilize them, and how individual differences shape these needs, remains underspecified. The present study addresses that gap.

### Goal and contribution of the present study

Previous research has established the general importance of psychological preparedness, treatment environment and trust in clinicians for therapeutic ketamine experiences, and has identified some environmental features, such as spiritual cues and music, as potentially beneficial. These findings have remained at a relatively high level of generality. The present study investigates these dimensions in greater detail and develops normative recommendations for how clinicians should design the treatment context to enable patients to feel safe in their choice for ketamine treatment.

Safety in this context has three senses that are easily run together, and the literature distinguishes being safe, an objective condition, from feeling safe, a subjective state that can come apart from it [24]. *Clinical safety* denotes the absence or adequate management of physiological and psychological risk; it is assessed against objective criteria, governed by protocol, and in this sense a matter of risk management [25]. For intranasal esketamine it is specified in regulatory terms: blood-pressure measurement before administration and at about forty minutes, observation until the patient is judged clinically stable, and a checklist-supported discharge decision [16, 26]. *Felt safety* denotes the patient’s experience of being secure enough to relinquish control during the acute phase; it is subjective, relationally constituted and individually variable, and a person may be safe without feeling so and feel safe without being so [27]. *Ethical protection* denotes what is owed to a person whose capacity for self-monitoring, articulation and refusal has been pharmacologically reduced by the intervention [28]. The three are related but independent. Clinical safety is necessary but not sufficient for felt safety, since a correctly monitored patient may still experience feelings of abandonment and hence feel unsafe. Felt safety is neither necessary nor sufficient for ethical protection, since a patient who trusts a clinician acting beyond what was agreed may, precisely because trust is high, be least able to register it. Felt safety therefore cannot serve as the criterion of adequate care, and where preference conflicts with monitoring requirements, clinical safety sets a floor beneath which preference cannot go. This study contributes to the understanding of felt safety, that is, of what is at stake above that floor in a treatment whose rationale requires the patient to relinquish control, and it does so through three analyses.

The study addresses three interrelated topics. First, we analyze patients’ pre-treatment concerns and expectations: what worries they have before treatment, how these affect the treatment experience, and what they would have liked to know in advance. The “last chance” motivation established in previous research may lead clinicians and patients to overlook concerns that, if addressed, could enable more realistic expectations, reduce anxiety and improve adherence; identifying them can equip clinicians to anticipate patient questions and tailor pre-treatment information.

Second, we investigate how specific clinician–patient interactions shape experiences of safety, conceptualizing treatment as a relationally embedded biopsychosocial process rather than a pharmacological intervention alone. Little is known about which concrete clinician behaviors patients experience as stabilizing or destabilizing during ketamine-induced altered states. Our analysis contrasts therapeutic presence and active physical grounding with destabilizing behaviors such as administrative distance, examines the post-acute transition and the role of structured transitional support, and relates patients’ interactional needs to different modes of experiencing safety, from reliance on medical authority to a need for relational resonance.

Third, we examine whether differing understandings of how ketamine exerts its effects relate to patients’ and clinicians’ contextual requirements. Many patients now arrive to treatment with pre-existing models, biological, spiritual or psychological, shaped by widely available information. We investigate whether these models are associated with different expectations of the treatment context, and whether clinicians’ and patients’ models are aligned, in order to clarify whether clinicians may benefit from adapting their approach to the individual patient’s framework even where it diverges from their own.

Two data sources inform these topics to differing degrees. The analysis of pre-treatment concerns and of the interactions and individual modes through which safety is achieved (the first two topics) rests on the patient interviews, since these topics concern the lived experience of treatment. The clinician interviews contribute to the broad thematic coding of the full corpus and are central to the third topic, in which patients’ and clinicians’ models of (es)ketamine’s mechanism of action and the associated contextual requirements are compared. Clinician perspectives therefore enter primarily as a comparative counterpart in that analysis, not as a parallel account of safety experience running through the whole study.

## Methods

This study is reported in accordance with the Consolidated Criteria for Reporting Qualitative Research (COREQ) [29]; a completed checklist indicating where each item is addressed is provided as Additional file 1. It forms part of the Taiwanese-German project “Psychedelic Transformations: A Taiwanese-German Comparison of Ethical and Sociocultural Aspects of New Therapies” (PsyTrans; https://www.psytrans.com/), which compares clinical and non-clinical uses of psychoactive substances, including (es)ketamine and psychedelics, in Germany and Taiwan. The data analyzed here are interviews performed in Germany with patients receiving intranasal esketamine (Spravato^®^) or IV ketamine infusion treatment and with clinicians administering these treatments. The study was preregistered on the Open Science Framework before data collection (https://osf.io/94rtw).

### Research team and reflexivity

Patient interviews were conducted by the postdoctoral researcher (AK) or by one of two psychology students serving as research assistants (JG, HS); clinician interviews by AK alone. AK holds qualifications in clinical psychology, psychotherapy and philosophy and held, at the time of the study, a three-year part-time research position funded by the PsyTrans project at a German medical university embedded in a clinical environment. JG and HS were female psychology students completing research internships and master’s theses; AK is male. All interviewers had previous experience in qualitative clinical interviewing.

No member of the research team had any prior personal or professional relationship with any interviewed patient; some clinicians were known to one researcher (DR) from professional contexts. Before each interview, participants received written study information setting out the purpose of the study and the interviewer’s interest in participants’ attitudes toward and experiences with (es)ketamine treatment, were informed verbally about it, and could ask questions before giving written informed consent. No researcher held a commercial or institutional stake in (es)ketamine treatment.

The PsyTrans project was designed by an international, interdisciplinary group of male and female researchers with German and Taiwanese backgrounds and expertise in medicine, anthropology, clinical psychology and philosophy; all share a professional interest in psychoactive substances, with an orientation toward their therapeutic potential ranging from agnostic to skeptical. Data collection, processing and primary analysis were carried out by researchers without experience of administering ketamine, who had not previously worked on the topic and held an agnostic stance toward its clinical usefulness (AK, JG, HS). During interpretation and writing, a male psychiatrist with clinical involvement in (es)ketamine treatment and research experience with psychedelics (DR) and two female researchers from Taiwan (KY, CN) joined the team, all three likewise agnostic. The team thus combined distance from the treatment during data collection, which may reduce pro-treatment bias, with clinical familiarity during interpretation; these positions were made explicit within the team and are reported here.

### Methodological orientation

The study employed structuring qualitative content analysis [30, 31] with an additional type-building analysis following Kuckartz and Rädiker [32]. The choice followed from the research questions rather than from practical considerations. Our questions are descriptive and comparative: what concerns patients bring to treatment, how safety is constituted in the treatment situation, and how patients’ and clinicians’ models of the treatment differ. Such questions call for a comprehensive, low-inference summary of the phenomenon in terms close to participants’ own, the domain of qualitative description [33], and qualitative content analysis is the strategy suited to it, since it applies defined research questions to a fixed corpus and organizes units of meaning into categories systematically enough to permit comparison across cases [34]. Theory-building approaches such as grounded theory pursue a different objective, the generation of an explanatory theory through greater transformation of the data and a higher level of abstraction [33, 34]. We sought a descriptive basis for subsequent normative analysis, not an empirically generated theory of safety.

A second consideration is decisive for an empirical-ethical study. The appropriate methodology depends on what the research is for, and where the aim is normative the empirical component must deliver what the normative analysis requires [35]. Our normative analysis (see Normative approach and From findings to recommendations) combines morally salient features of the treatment situation with independently justified ethical premises, which requires that each finding remain traceable to the accounts it derives from, so that the step from finding to recommendation can be inspected and contested. A theory generated from the data would interpose a further layer of researcher abstraction and make that inspection harder. The type-building analysis of mechanism-of-action models (see Data analysis) is the one component that moves beyond description, since that question concerns patterns across cases; it is conducted within the same framework [32] and its results are reported as provisional interpretive patterns rather than theory.

#### Normative approach

Empirical findings do not by themselves establish obligations. What participants report identifies features of the treatment situation that are morally salient; normative conclusions follow only when such findings are combined with independently justified ethical premises. The section (From findings to recommendations) makes each such combination visible, setting out for every recommendation the finding, the ethical consideration invoked, the reasoning connecting them, the considerations against, and the resulting strength (Table 2).

The ethical premises we use are drawn from the analysis of vulnerability developed in feminist and relational bioethics [36, 37]. This framework fits the research interest because felt safety, as defined in the section (Goal and contribution of the present study), is the patient’s experience of being secure enough to relinquish control, and the question of what is owed to a person who has relinquished control into another’s care is a question about vulnerability. We adopt it rather than a principlist framework because the morally decisive feature of the situation is a dependency that the intervention itself produces: a framework organized around vulnerability names this feature directly, whereas one organized around the balancing of general principles registers it only indirectly, as a factor bearing on autonomy or beneficence.

Two elements of this literature serve the purpose of this study. From Luna we take the view that vulnerability is not a label attached to a population but a set of layers, each arising from a specific relation between a person and a circumstance and each removable in principle. Layers are dispositional: they are latent susceptibilities that become actual only when a stimulus condition triggers them, so that an analysis must identify, for each layer, what triggers it, who controls the trigger, and which responses relieve it. Some layers are cascading: they generate further layers, as when a first harmful experience produces distrust that then prevents later needs from being voiced [37, 38]. From Rogers, Mackenzie and Dodds we take the distinction between inherent vulnerability, which belongs to embodied human life, situational vulnerability, which arises from a person’s circumstances, and pathogenic vulnerability, which is generated or worsened by the very responses meant to address it, including paternalistic ones; and the requirement that responses to vulnerability engage and restore agency rather than replace it (for a review of taxonomies see [36, 39]). Luna’s term cascading covers Rogers’ pathogenic layers without implying ill intent, since such layers can arise from ordinary institutional routine [38]; we use it for that reason.

How these elements generate obligations needs stating, because it has been objected that vulnerability has no normative force of its own, and that whatever normative force it appears to have in fact derives from other, more basic concepts, such as need, dependence or consent [40, 41]. We do not claim otherwise. The normative weight of our conclusions rests on three premises that are defensible independently of the concept. First, the acute state reduces the patient’s capacity to monitor, articulate and refuse, and so produces a dependency on those present. Second, the party that produces this dependency is the party on whom the patient then depends; special responsibilities attach to a party when someone else has been made vulnerable specifically to that party’s conduct [28, 36], and these responsibilities are strengthened rather than weakened where the dependency was foreseeably brought about by that party. Third, the clinical role already carries fiduciary obligations of care toward the patient [42]. These three premises carry the entire normative weight of the argument. The vulnerability analysis is not a further, fourth premise added to them, and it contributes no independent normative force of its own. Its role is diagnostic: for a given situation it specifies which layer of vulnerability is at issue, when that layer is triggered and by whom, and which responses relieve it and which deepen or propagate it. In doing so it determines what the three premises require in the concrete case, without adding anything to them. This is how the analysis is used throughout the Discussion and in the section (From findings to recommendations).

### Participant selection and setting

Participants were recruited through convenience sampling with a snowball component. Patients were recruited at cooperating clinical sites where they received (es)ketamine treatment, clinicians from these institutions or from the researchers’ professional networks; both were invited to refer further potential participants. Because the cooperating sites informed the team only of patients willing to be interviewed, the number of patients who declined was not recorded.

Patients were informed about the study by clinicians at the cooperating sites, who passed on the research team’s contact details; patients then approached the team or agreed to be contacted by it. Participation was voluntary, had no consequence for treatment, and was not compensated. Because sites forwarded only the details of willing patients, participation was visible to the informing clinician whereas refusal was not communicated by the research team; no researcher held clinical responsibility for, or was involved in the care of, any participant. The safeguards for voluntariness and the procedures in place in case an interview caused distress are described in Additional file 2, section A; no interview had to be interrupted and no participant required support afterwards.

The analyzed sample consists of 24 participants: 14 patients who received ketamine treatment, 9 with esketamine (Spravato^®^) and 5 with IV ketamine infusions, and 10 clinicians involved in such treatments. Of the 14 patients, 7 were female and 7 male; of the 10 clinicians, 8 were female and 2 male. No participant was excluded; all 24 interviews were transcribed, coded and analyzed. The three analytic components address different parts of the corpus, the first two the patient interviews and the third the full corpus of patients and clinicians; within each component every eligible case is included, and no case was omitted for want of codeable material (see Data analysis). Patient interviews were conducted in person or online according to preference, clinician interviews online, all one to one with no one else present.

Beyond these descriptors, participant-level demographic and clinical data were not systematically collected. This was a design decision: the study was not intended to stratify findings by patient characteristics but to capture the range of experience across the treated population, and data minimization was applied in view of the sensitivity of the clinical group and applicable data-protection requirements. The consequences for transferability are addressed in the Limitations section.

All patients were treated for treatment-resistant depression as outpatients, in the two contexts described in the section (The two treatment contexts), and were aged between 27 and 66 years at interview. All were interviewed during or after their course of treatment, so that every patient had direct experience of the substance; interviews were arranged as soon as access to willing participants could be obtained rather than at predefined points in the treatment course, because our questions concern the treatment situation as such, for which we did not regard the precise point in the course as decisive provided the acute experience had been undergone. The clinicians comprised all professional groups involved in direct patient care during these treatments, including physicians, psychiatrists, psychological psychotherapists and nursing staff. Recruitment and data collection began after ethics approval in 2024; interviews were conducted between 20 March 2025 and 10 February 2026. Data collection is complete, and the analysis reported here draws on all interviews conducted in that period.

The following were not collected and cannot be reported: the number or range of treatment sessions per patient; the interval between the acute phase and the interview, and the number of patients interviewed during as opposed to after treatment; the length and nature of clinicians’ relevant experience; and the number of patients treated or approached at the cooperating sites during recruitment. Clinicians and patients were not matched, and the recruiting institution was not recorded in the analyzed dataset.

#### The two treatment contexts

Participants were drawn from two clinically distinct contexts. Both involve supervised administration of a substance with acute consciousness-altering effects, but they differ along dimensions potentially relevant to the experience of safety, summarized here and described in full, with the regulatory and procedural detail, in Additional file 3.

Intranasal esketamine (coded S) has been approved in Germany since March 2021 for treatment-resistant depression as an add-on to an oral antidepressant and is covered by statutory health insurance [16, 43, 44]. The patient self-administers it from a single-use device under direct supervision, retaining a degree of physical control over intake; administration is non-invasive and brief and is followed by an observation period governed by physiological monitoring and ending in a checklist-supported clinician judgement that the patient is clinically stable [16, 26, 45]. Patients receive the treatment in a health care facility repeatedly (usually once or twice a week) over months on a standard schedule. Although the informed consent process accounts for all potential effects of the substance, including its acute consciousness-altering effects, neither the regulatory framework nor the manufacturer’s mandated materials provide for psychological preparation for the acute experience or its subsequent processing; the end of a session is defined as a discharge decision taken by the clinician. The esketamine patients interviewed here were treated at hospital outpatient clinics.

Intravenous racemic ketamine (coded IVI – intravenous infusion) is used off-label for the same as well as other indications. The German National Care Guideline advises against its use outside inpatient psychiatric settings and names the risk of commercially motivated provision as a privately billed service [46]; the patients interviewed here nonetheless received it as outpatients at several sites and paid for it themselves. Administration is more invasive, requires venous access and is controlled entirely by the clinician, so that the patient cannot interrupt or modulate intake; the acute phase is (depending on the infusion) in most cases longer than for the nasal spray and is followed by an extended monitored rest period [44, 47]. Because the treatment is off-label there is no binding protocol and practice varies locally; we therefore report the reference protocol from the German practice literature (Additional file 3) rather than documented site practice, and treat what patients reported about dose, setting, stimulus regulation and accompaniment as data. All patients in this group received some form of integration work alongside the infusions, differing between individuals and sites.

The contexts thus differ in route and invasiveness, in patient control over intake, in duration of the acute phase, in regulatory status and monitoring, in who bears the cost, and in the presence of an integration component. They also differ in internal homogeneity: the esketamine arm operated within a single licensed framework, the IV infusion arm across several sites without a common protocol. We return to the consequences in the Limitations.

### Data collection

Interviews were semi-structured, conducted in German or English, and based on a guide developed for the study, which was piloted by the Taiwanese and German teams with one interview each; as the guide was not substantively changed, the German pilot interview was retained in the sample. The guide is published with the preregistration (https://osf.io/94rtw) and had not been used previously; the sections used here, with the questions put to patients and clinicians, are reproduced in English and in the German wording used as Additional file 4.

Interviews were audio-recorded using Renkforce recording devices (Conrad Electronic SE). No field notes were made during or after interviews. Clinician interviews lasted between 24 and 62 min (mean 38 min), and patient interviews between 23 and 134 min (mean 42 min).

The preregistration set a target of 17 interviews per group, derived from a systematic review of saturation in comparable studies [48]; it was not reached in either group, data collection being governed by access to willing participants rather than by an analytic stopping rule. No systematic assessment of saturation was carried out and we do not claim thematic saturation. We claim instead that the sample provides sufficient information power [49] for exploratory, descriptive aims in a narrow study population, with dense topic-specific material and an analysis that applies established frameworks rather than generating theory; information power is weakest for the clinician sample and for the smaller of the two patient groups, the five patients treated with IV ketamine infusions (compared with nine treated with intranasal esketamine). The basis of the original target, the criteria we did not apply [50], the distinction between code and meaning saturation [51], and the reasoning behind the information-power claim are set out in Additional file 2, section B, and the consequences in the Limitations.

Audio recordings were transferred to secure institutional computers, deleted from the devices, and transcribed with the locally operating open-source software noScribe. Transcripts were proofread and anonymized manually within a few days of each interview, after which the audio files were deleted and the anonymized transcripts saved to a project database accessible to the team. Selected excerpts were translated into English for publication, with original German terms in brackets where needed to preserve nuance. Transcripts were not returned to participants for comment or correction.

Informed consent forms and participant lists are stored in the institution's archive in accordance with German legal requirements. The study was approved by the ethics commission of Charité Berlin (EA4/176/24), and all procedures adhered to the Declaration of Helsinki.

### Data analysis

The analysis was staged to match the three research aims, which differ in the kind of knowledge they require. All procedures were drawn from the integrated framework of qualitative content analysis [30–32], ensuring shared epistemological assumptions and a single software environment (MAXQDA, version 24.11.1) throughout.

The first aim, mapping the breadth of patients’ concerns and experiences, called for category-based structuring content analysis, which orders thematic content and permits comparison across cases; it drew on the 14 patient interviews.

The second aim, understanding how safety is relationally constituted within individual patients, required moving from categories to whole cases. We differentiated the safety-relevant categories inductively, synthesized them case by case, and condensed them into overlapping interpretive patterns, an approach that captures patterns a purely category-based reading would fragment; it drew on all 14 patient interviews; how the inductive subcategories were developed and applied to the full patient corpus is described below in this section (see also Additional file 8).

The third aim, whether patients’ and clinicians’ understandings of ketamine’s mechanism of action are associated with differing requirements of the treatment context, concerns each person’s overall stance toward the treatment and was addressed through type-building content analysis, which groups cases into types defined by a shared set of features; it drew on all 14 patients and 10 clinicians. This progression from category-based to case-based to type-based analysis follows the levels of abstraction described in Kuckartz’s approach and allowed each question to be analyzed at the appropriate grain.

The category system was developed deductively and inductively. An initial system of 623 codes was developed by the Taiwanese team members (KY, CN) from the PsyTrans framework and the interview guide, designed to cover both national contexts (Additional file 5). It was piloted on German and Taiwanese material and reduced in a group consensus process on conceptual grounds rather than by frequency alone: codes were merged where content overlapped, removed where rarely or never applied, and recalibrated so that each category corresponded to a meaningful unit of analysis. The resulting 20 main categories (Additional file 6) were applied to the entire corpus. Themes were thus partly specified in advance and partly derived from the material.

In the first stage the entire material was broadly coded along the 20 main categories, assigning at most five categories per interview paragraph to preserve conversational context.

Intercoder agreement was established before the study material was coded. The three researchers who coded the data (AK, HS and JG) calibrated against one another in three rounds, in each of which all three independently coded one complete interview transcript with the full category system; the three transcripts were drawn from the 24 study interviews (12.5% of the material). Discrepancies were discussed after each round and used to sharpen category definitions, so that the procedure stabilized the category system as well as documenting agreement. Agreement was calculated pairwise with the Intercoder Agreement function of MAXQDA at segment level, with a minimum code overlap of 90% so that trivial differences in segment boundaries were not counted as disagreements, and is reported as percentage agreement rather than as a chance-corrected coefficient. The target of a mean agreement of 80% across all codes was reached on the third transcript, after which coding of the remaining material began. Following calibration, each component was coded by a single researcher: pre-treatment concerns by AK, safety experience and clinician–patient interaction by JG, and mechanism-of-action models by HS. No separate coefficient was computed for the inductively developed subcategories, which were differentiated from already calibrated main categories; consistency was maintained through repeated comparison against the written codebook (Additional file 7), which gives for every category its definition, derivation, coding rule and an anchor example.

In the second stage, eleven of the 20 main categories were selected for further inductive differentiation, against the research questions and before coding frequencies were inspected; they are marked in Additional file 6. All segments belonging to them were retrieved with the MAXQDA text retrieval function and differentiated into 36 inductive subcategories formulated directly from the material, with linguistic imagery and metaphors treated as analytically significant units of meaning.

Two of these categories carry the analysis of safety reported here: Roles of or Relationship with Healthcare and Medical Professionals, and Influences of Treatment Environment. Taking main and subcategory codings together they account for 19.2% and 12.3% of the coded material and hold 15 of the 36 subcategories. A third category, Evaluation of Psychedelic Treatment, is marginally ahead of the second (12.4%) and the most frequent when main categories are counted alone, but was differentiated into only two subcategories and concerns retrospective evaluation rather than the constitution of safety; it was therefore not prioritized. Frequencies are context, not statistical weighting; the full distribution is given in Additional file 8.

Data collection ran in parallel with analysis, and the components were begun at different points in recruitment. The 20 main categories were applied to all 14 patient interviews. The 36 subcategories were developed on the interviews available when that component was begun and were then applied to the three interviews completed subsequently, so that all 14 patient interviews are coded at both levels and the findings in the Results section (The role of clinician-patient interaction in shaping safety) rest on the complete patient corpus. The type-building analysis of mechanism-of-action models likewise draws on the full corpus of 24 interviews (Additional file 9). No analytic component therefore rests on a subset of the cases eligible for it; the counts reported are those of the full corpus addressed by each component, and Additional files 8, 9 and 10 document the distribution of codings and the composition of each component.

In a final step the findings from these categories were synthesized into thematic case summaries and condensed into three modes of experiencing safety. These modes are overlapping interpretive patterns derived from the case summaries, not mutually exclusive classifications of participants: they were not produced by formal type construction, and most participants combine features of more than one. This distinguishes them from the typology of mechanism-of-action models described next.

The typology of mechanism-of-action models was developed following Kuckartz and Rädiker’s type-building qualitative content analysis [32], within a feature space of five dimensions: mechanistic understanding, clinical role and value, experiential significance, efficacy assessment, and patient agency. Cases were sorted polythetically, by overall similarity across dimensions, into three types, each as homogeneous internally and as distinct from the others as possible and each comprising eight cases with varying proportions of patients and clinicians. All 24 participants could be characterized across the five dimensions and entered the typology. To relate the models to what participants needed from the treatment context, we then drew on the system’s five setting-related categories: preparation, environment, clinician presence, the post-treatment transition and post-session processing.

No formal methods of enhanced trustworthiness such as member checking, peer debriefing, or independent audit of the coding process were employed; in particular, participants did not provide feedback on the findings. The trustworthiness of the findings rests on the transparency of the analytical process, the systematic and iterative development of the coding system, the use of an established methodological framework, and the documentation of researcher positionality and reflexivity reported above. In the Results section, participant quotations are presented to illustrate each theme and type, and every quotation is identified by an anonymized participant code.

The analysis of safety experience was conducted at the level of the individual case. Categories were differentiated across the patient corpus, synthesized case by case and condensed into interpretive patterns; treatment modality was not a sorting criterion at any stage, and the resulting modes are defined by relational patterns rather than by the treatment received. We report for the central findings whether they were articulated in both treatment contexts, but not frequencies by modality, since with nine and five patients in each modality the absence of a theme in one group would be uninformative.

Three departures from the preregistration should be noted. First, the preregistered 623-code system was reduced to 20 main categories before the analysis proper; the preregistration anticipated iterative refinement but not a reduction of this magnitude. Second, the modes of experiencing safety are reported as overlapping interpretive patterns rather than as the result of a formal type construction. Third, and most substantially, the analysis of mechanism-of-action models does not rest on the 20-category system: on review those categories proved insufficiently aligned with that question, and a separate system of six main categories was developed inductively for this component, one capturing models of the mechanism of action, differentiated into the five dimensions of the feature space, and five organizing setting-related material, each subdivided by whether a statement concerned the relevance of a feature or its implementation. This system, with the distribution of codings for all 24 participants entering this component, is given in Additional file 10.

The mechanism-of-action types are reported as provisional interpretive patterns rather than validated classifications. Types were constructed polythetically, so that no case matches its type on every dimension; cases combining features of more than one type were assigned on the balance of dimensions and, failing that, on the mechanism-of-action dimension; classification was carried out by HS and reviewed against the coded material with AK, with divergent judgements resolved by returning to the case summaries; and cases that could not be characterized across the dimensions would have been left unassigned rather than allocated to the nearest type, although in the event no case required this. The labels are shorthand for configurations of features, not clinical profiles or characterizations of individuals. A de-identified case-by-dimension matrix, the case-to-type assignment and the distribution of patients, clinicians and modalities across types are given in Additional file 9; the provisions are stated in full in Additional file 2, section C, and apply more strongly to the modes of experiencing safety, which are overlapping interpretive patterns rather than types.

### Use of large language models

Large language models were not used in data collection, transcription, coding, category development, typology construction, the derivation of the modes of experiencing safety or the selection of quotations. No identifiable participant data were entered into any model at any stage, nor were any research data, that is, transcripts, audio recordings, coding files or the MAXQDA project; the dataset is held on secured local servers of Charité – Universitätsmedizin Berlin. In preparing the revised manuscript and the additional files the authors used a large language model (Claude, Anthropic) for language editing and for drafting and restructuring passages of text. The material entered into the model consisted solely of manuscript text, including anonymized quotations already selected by the authors for publication; the responsible data protection officer confirmed that this does not constitute processing of personal data. All analytic decisions, normative positions and the final text are the authors’ own, who take full responsibility for the manuscript. The model is not an author.

## Results

Throughout this section, participant quotations are identified by anonymized codes. S denotes patients treated with esketamine nasal spray (Spravato^®^); IVI denotes patients treated with intravenous ketamine infusions accompanied by structured integration work (a difference in accompanying care addressed in The two treatment contexts and in the Limitations); and E denotes interviewed clinicians (experts).

### Concerns regarding ketamine treatment

Our analysis examined patients’ concerns at three stages: before treatment, during treatment, and in retrospect. Their motivations were largely consistent with previous findings, as most viewed ketamine as a last resort for their treatment-resistant depression. Their worries, however, were more varied than this shared motivation might suggest, and fell into several distinct patterns that persisted even alongside their strong wish to be treated.

#### Concerns before treatment

Three patterns of pre-treatment worry emerged.

- **Type 1: Acute effect worry.** A recurring concern was the fear that ketamine would produce LSD-like or psychedelic effects, sometimes linked to previous substance experiences and to the worry of developing lasting psychological problems such as psychosis:
  - *IVI_05: I was definitely afraid that it [Ketamine] would have an LSD-like effect.*
  - *IVI_05: I was afraid to get a psychosis. I had this when I took LSD, very very bad. I mean my LSD trip was horrible.*

For some, such fears were only partly mitigated by the clinical setting and remained distressing.

- **Type 2: Side-effect framing**. A further pattern was the framing of psychoactive effects as side effects rather than as part of the therapeutic action. This reframing appeared to serve an emotion-regulating function: if the effects are merely side effects, they are possible but not threatening. S_01, for instance, focused on hope while minimizing the experiential dimension:
  - *S_01: “[...] it is rather the hope I try to keep in mind and everything else that happens are more or less side-effects during the treatment, and you know that you are good for nothing the rest of the day, you don't feel well, and that's it.”*

A patient who had asked their clinician not to say more than necessary later reflected that this had left them unprepared:

*S_04: “Perhaps I could have asked for more details*,* because the side effects have been rather unpleasant the first time.”*

A third patient (S_03) used the same framing while acknowledging mild concern about dissociation.

- **Type 3: Absence of worry**. A third subgroup reported entering treatment without notable concern, which is striking given that several of them described worry as a general tendency. One actively limited information intake to avoid overthinking:
  - *S_01: No, I intentionally did not inform myself in detail. I told my Dr. she shall only tell me the most important things roughly, so that I don’t overthink it.*

For another, the fact that the treatment was covered by health insurance, strongly associated in the German system with evidence-based care, was itself reassuring:

*S_03: No*,* not at all*,* since I knew that the health insurance pays*,* which strangely makes me less anxious*,* although I usually tend to worry about things.*

Others described a more dispositionally open or pragmatic stance, taking the experience as it came.

#### Concerns during treatment

Asked whether their pre-treatment worries persisted, most patients reported no treatment-specific concerns but a continued preoccupation with pre-existing general worries:

*S_09: No*,* I mean I carry them [worries] with me all the time. During the session I rarely have them.*

A few were relatively worry-free, an attitude combined with an open stance toward the experience:

*S_03: During it [the treatment] not really*,* well I knew that is a state that will pass and so it was kind of funny.*

More specific concerns were also reported, particularly the persistent fear of psychosis, and one patient described earlier traumatic experience in a clinic setting resurfacing during administration:

*IVI_05: Generally it was the fear that I always had and still have after the treatment*,* that it [the treatment] remains uncomfortable or I develop a psychosis.*

*S_04: I had traumatic experiences in a clinic and they come up at the moment when I take Spravato. They are also there in everyday life*,* but when I take Spravato this somehow causes my fears and traumas to come up.*

Others were preoccupied not with fears but with hopes, particularly the expectation of meaningful insight:

*IVI_02: Yeah*,* I was waiting to see how this is gonna help me*,* how it was gonna make me feel better. And I also thought maybe I can find answers there to the questions I had at the time.*

#### Information patients would have liked to receive before treatment

Asked in retrospect, most patients reported having been sufficiently informed (IVI_05: “No, I do not think so”). A striking contrast was IVI_01, who reported a broadly negative experience of preparation, setting, and support, and who wanted information that would normally form part of standard pre-treatment education, suggesting it had been inadequate in their case:

*IVI_01: I think generally it would have been good if someone would have explained the procedure to me. So generally what will happen*,* that I lay down there*,* what can happen*,* the amount*,* how long the trip will last*,* and after that we do this and that. Just really a detailed*,* structured explanation of what will happen to me. […] Or what may happen in terms of severe side effects*,* of course without horror stories. And also how the integration works. So also to answer questions that I ask also about the integration session. That you sit down*,* that we talk about it*,* these things have to be explained.*

### The role of clinician-patient interaction in shaping safety

Interpersonal interaction was described by participants as central to their sense of safety, experienced along a continuum from technocratic-functional care to relationally responsive support.

#### Therapeutic presence versus administrative distance

A key distinction was between mere physical attendance and therapeutic presence. Participants described sustained, non-verbal presence not just as a comfort but as a functional prerequisite for “letting go,” a stabilizing “secure base” that enabled them to relinquish control without fear of being overwhelmed:

*IVI_05: The psychiatrist sat directly next to me… he gave me safety… he calmed me down and took away my worries.*

In contrast, administrative distance, particularly clinicians using laptops or checking emails during sessions, was experienced as a significant rupture, intensifying feelings of abandonment and leading participants to describe the treatment as a dehumanized, “industrial” process:

*IVI_01: He sat there the whole time at the laptop… I think he also checked some emails… [I had to protect myself from the] abyss*,* that the therapist is really not there.*

A purely procedural presence, limited to physical assessment, was experienced similarly:

*S_06: I hold out my arm*,* he takes my blood pressure*,* does this to me*,* and that’s it. Other than that*,* there’s no interaction.*

#### Active grounding and expertise on equal footing

During ego-dissolution or intense anxiety, participants described a strong need for active physical grounding. Hand-holding emerged as an essential “external reference” (Referenz im Außen), providing orientation when internal structures were perceived to collapse:

*IVI_02: The first time I asked him to hold my hand because I was like: ‘Do I exist still?‘… Oh*,* there is a body there somewhere. I can feel it.*

Participants also emphasized “expertise on equal footing.” Transparent, non-hierarchical communication mattered especially to those with prior knowledge, who felt alienated by hierarchical distance, dismissive or ego-driven communication, or perceived incompetence. Some patients wished their clinicians had personal experience of the substance, and one patient lost trust after a sarcastic response to his technical questions:

*IVI_01: ‘You’ve certainly done your research*,* you’re a real expert’.*

Others felt reassured simply by long familiarity with the facility and its staff:

*S_03: I’d known them all for quite some time and so I actually felt quite confident about them.*

#### Integration and the need for “landing aid”

The post-acute phase emerged as a particularly sensitive transition. Many participants described the abrupt end of sessions as being “spat out” (ausgespuckt) into a hectic environment or “discharged by the clock” (nach der Uhr eingestellt und entlassen):

*S_09: [The transition was] very hard… [like being] spat out (ausgespuckt) [into the hectic world]… [I wish for a] softer landing.*

This felt lack of integration is further corroborated by two other patients. Both emphasize a gap between the acute experience and its psychotherapeutic processing. One highlights that on-station time constraints made clinical integration nearly impossible, noting:

*S_08: There just wasn’t enough time for that. I think I only saw the [therapist] twice.*

Similarly, another person articulates a desire for structural embedding:

*IVI_04: This really being embedded [in] the treatment…. So perhaps actually discussing it afterwards every single time…*,* as I did during the ketamine therapy*,* what I experienced.*,* that’s what I would have wished for.*

This was contrasted with the positive impact of ritualized reintegration, such as a brief debriefing or shared tea:

*IVI_05: .drinking tea together after the therapy… that gave one a feeling of well-being.*

To conceptualize this unmet need, we introduce the notion of “landing aid”: a structured, ritualized transition process that facilitates the safe integration of acute experiences before re-engagement with everyday contexts.

#### Modes of experiencing safety

The experience of safety was not uniform but shaped by distinct relational patterns, which we describe as three modes of experiencing safety.

These modes are overlapping interpretive patterns derived from case summaries, not mutually exclusive classifications of participants: they name recurring patterns in how safety was constituted, most participants combined features of more than one, and the labels are not descriptions of persons or validated clinical profiles. For this reason no case-to-mode assignment is reported (Additional file 9, section C); the illustrative cases below indicate which accounts most clearly exemplify each pattern.

For the *informed skeptic*, safety is tied to intellectual control, technical expertise, and transparent, non-hierarchical communication. These participants often hold an “expert-patient” identity, using precise neurobiological terminology to categorize and de-pathologize their experience:

*IVI_01: My understanding was actually that psychedelics… increase the brain’s neuroplasticity… essentially enabling synaptic connections…so to speak… thereby dampening the default mode network*,* calming the amygdala… and also the glutamate system…*.

This type is highly sensitive to disruptions in the alliance and to a technocratic-industrial style of care, and may feel dehumanized when treatment is reduced to a procedural minimum or when a clinician is perceived as asserting hierarchical distance (metaphorically, “the only rooster in the yard,”):

*IVI_01: I felt like I was in a machinery*,* on a conveyor belt… I am a product being processed here.*

In contrast, the *relationship-oriented truster* derives safety from interpersonal resonance, emotional warmth, and the continuous presence of the clinician. Here medical authority and institutional structure are not distancing but reassuring, a solid framework for the process:

*S_05: That feeling of medical authority gave me kind of like a reassurance basically.*

These individuals find stability in the professional setting, where monitoring of vital signs or the clinician’s expertise serves as a surrogate for safety, and even institutional signals such as insurance coverage can validate legitimacy. For this type, safety is constituted through interpersonal warmth, with the clinical staff seen as humanized, approachable partners:

*S_08: I had a really nice care worker there… and we’d sit there sometimes*,* it was really good fun*,* and had a laugh.*

*IVI_05: [The psychiatrist was so human and approachable that I could imagine] having a beer with him.*

Finally, the *autonomous self-regulator* actively regulates safety internally, valuing clinicians mainly as a background safety structure; excessive interaction during the acute phase may feel intrusive. Safety is established through individual strategies such as eye masks, earplugs, or curated music, and through personal rituals or trusted companions brought into the room:

*S_01: With my two female friends*,* I did it that way*,* that they are in the treatment room… That was also like an additional safety pillar.*

The findings that carry this study’s central claims were articulated in both treatment contexts. The distinction between therapeutic presence and administrative or procedural distance was drawn in both (the laptop account comes from the IV infusion context, the account of contact reduced to a blood-pressure measurement from the esketamine context), as was the need for a structured post-acute transition (being “spat out” was reported by an esketamine patient, the counter-example of shared tea by an IV infusion patient). Concerns about acute effects, and the persistence of general rather than treatment-specific worry during sessions, were reported in both groups. Both the most negative account of preparation, presence and integration and one of the most positive accounts of relational accompaniment come from the IV infusion context, in which integration work was provided; a structured integration component did not, in these cases, determine whether treatment was experienced as safe. Two findings are exemplified only through IV infusion patients, the informed-skeptic mode and hand-holding as physical grounding, and should be regarded as provisional.

#### Divergent and negative cases

Several accounts ran counter to these patterns. Not all participants sought relational presence: one patient experienced clinician interaction during the acute phase as intrusive, preferred to regulate the experience alone with eye mask and music, and treated institutional and procedural cues rather than persons as the source of security. The need for a structured transition was not universal: some described the end of the session in neutral terms and wished for no closure. Physical grounding was valued by those who described it but not raised by most, and we cannot establish whether it was unwanted, not offered, or not salient in interview. The strongest counter-case to any simple association between structured care and felt safety is that the most negative account of accompaniment comes from the context with structured integration work. Finally, one participant declined further treatment because the environment was too disruptive. These cases bound the claims that follow: the patterns organize variation in this sample and are not uniform features of the treatment. Their bearing on the ethical analysis, which treats such variation as expected rather than anomalous, is taken up in the Discussion (Modes of experiencing safety).

### Models of mechanism of action in patients and clinicians

The analysis revealed substantial differences in participants’ models of how ketamine works in depression. On that basis, three types were developed, each comprising eight of the 24 participants (see Data analysis). Notably, most interviewed clinicians belonged to Type 1, whereas patients were more often classified as Type 2 and Type 3.

All 24 participants were assigned to a type (Additional file 9). Type 1 comprises 6 clinicians and 2 patients (1 IV ketamine infusion, 1 nasal esketamine), Type 2 comprises 2 clinicians and 6 patients (4 IV infusion, 2 esketamine), and Type 3 comprises 2 clinicians and 6 esketamine patients. Clinicians are thus concentrated in Type 1 (see Discussion, Models of mechanism), and no IV infusion patient was classified as Type 3, which we report descriptively and do not interpret as a modality effect, since IV infusion treatment is confounded with integration work and self-payment (see The two treatment contexts and Limitations) and with five IV infusion patients the absence of a type is uninformative. As with the safety modes, these types are provisional interpretive patterns, constructed polythetically, and the labels are shorthand for configurations of features rather than characterizations of participants.

- **Type 1: Catalyst of transformative processes.** Ketamine is conceptualized as a catalyst for profound, potentially lasting transformation. Subjective experiences are inherently meaningful, facilitating insight, neuroplastic change, and active restructuring of cognitive, emotional, and behavioral patterns, with patients as active agents:
  - *E_07: (...) Some tell me directly afterward that they feel they've just experienced something helpful: 'Finally, I could examine it, though it was very difficult at times'.*
- **Type 2: Moments of clarity and relief.** Vague neurobiological assumptions combine with experiential interpretation. Ketamine is a limited but valuable option associated with temporary relief or transient insight, valued mainly for its acute effects; active engagement is not essential but openness to reflection is beneficial:
  - *IVI_05: (...) I have these obsessive thoughts or unpleasant thoughts, or self-doubt, self-hatred. All of that was gone. It was as if it didn't even exist. It didn't matter, it just wasn't important. And it was just so wonderful, because you could kind of breathe deeply, as if nothing were wrong, everything is fine after all. And anything can be, and nothing has to be, that was a saying I took with me (...) it felt like a profound sense of freedom.*
- **Type 3: Pharmacological symptom management.** A predominantly biomedical understanding of ketamine as a pharmacological intervention for symptom reduction. These participants described its mechanism only in simplified or unspecified terms and did not consider a detailed understanding of how it works relevant to their treatment. Subjective experiences were framed as side effects without intrinsic meaning, and, where patient agency was addressed, patients as relatively passive recipients:
  - *S_01: (...) I'm going to a 'Head Cinema' event (...).*

The contextual expectations participants articulated appeared related both to shared treatment-related needs and to these underlying models. Type 1 participants expressed more differentiated expectations and more critical evaluations of practice, alongside greater openness to self-initiated coping and integrative practices. Type 3 participants more often described enduring the treatment without raising difficulties, articulated fewer specific expectations, and less often raised preparation, processing, or environmental conditions, while nonetheless reporting anxiety. We report this as a difference in what participants articulated in interview and not as a characterization of their engagement with their own care; alternative readings of the difference are considered in the Discussion (Models of mechanism). Type 2 participants occupied an intermediate position.

A recurring theme was the tension between “letting go” and control: among Types 1 and 2, letting go was therapeutically meaningful and associated with emotionally salient or insight-generating experiences, while participants in Types 2 and 3 who had agreed to treatment reluctantly feared losing control. Surrender was understood as an active process presupposing trust and support rather than as passive submission.

The greatest differences across types concerned preparation and post-session processing. Participants with experiential, process-oriented models (mainly Type 1, partly Type 2) treated these as fundamental, emphasizing intentionality, collaborative interpretation, and patient involvement; where clinicians focused on intentions and insights, patients mainly hoped to share, process, and interpret their experiences, and felt left alone when this was lacking. Type 3 participants attributed little importance to these components, finding brief follow-up sufficient:

*S_08 (Type 2): If I could wish for something*,* it would have been nice to have more time afterward to go deeper*,* to look more closely. What was happening there? Why that specifically?*

*E_03 (Type 1): So it’s about stabilizing this long-term. And that doesn’t happen by simply giving ketamine and leaving them alone with it*,* but rather by activating resources and truly helping them regain their strength.*

Across all types, participants associated treatment with heightened sensitivity and valued calm environments, sufficient transition time and emotionally available clinicians; emotional absence or disinterest was described as more distressing than temporary physical absence, whereas several clinicians stressed constant physical presence. Type 3 participants associated a quiet environment primarily with safety and stability, Types 1 and 2 with engagement with the experience; one Type 1 patient declined treatment because the environment was too disruptive.

Resource limitations were reported to constrain individualized support and supportive environments, to which participants described themselves as especially sensitive under ketamine. Awareness of clinical workload intensified guilt and fear of being burdensome and reduced willingness to articulate needs, whereas attentive behavior and small gestures of personal concern were experienced as substantially mitigating structural limitations:

*IVI_04 (Type 2): I suppose it’s also quite a lot of work for them at the hospital. Especially when it’s intravenous*,* the question was always who would do it (…) I did get the feeling that it was somehow difficult for all the doctors (…) I’m not the sort of person who says*,* ‘Oh*,* but I’d still like to have it.’ That’s not me.*

*S_01 (Type 3): I would wish for different circumstances*,* but I see that (…) they are doing what is within their power. (…) when I was there during winter time*,* I always was offered tea (…) So I don’t need a blanket and a bed in that sense*,* but just this relaxation*,* recovery. And the people there do that as well as they can*.

Across the types, several needs were articulated independently of the mechanism-of-action model, while the perceived relevance and function of individual treatment components appeared linked to whether ketamine was understood as a pharmacological intervention or as a catalyst of an experiential and relational process. The implications are drawn in the Discussion (Models of mechanism) and the recommendations in Recommendations for clinical practice.

Table 1 summarizes the themes and types reported above, the evidence on which each rests, and the implication we draw from it. The implications are stated for orientation; the reasoning supporting each, the considerations against, and the strength of the resulting recommendation are set out in From findings to recommendations and Table 2.

**Table 1:** Summary of themes, interpretive patterns and types, illustrative evidence, and proposed implications

Theme or type	Empirical content	Illustrative evidence	Proposed implication (see Discussion)
Pre-treatment concerns	Three patterns: worry about acute psychoactive effects; reframing of those effects as side effects; information avoidance as anxiety management. Concerns persist alongside a strong motivational pull.	Accounts of fear of loss of control; description of dissociation as a side effect to be tolerated; deliberate avoidance of detailed information before the first session.	Preparation addressed to the experiential dimension, not only to factual information (Pre-treatment concerns; Normative pathway; recommendation R1).
Therapeutic presence versus administrative distance	Physical attendance distinguished from attentive presence; administrative activity during sessions described as rupture. Reported in both treatment contexts.	Account of a clinician working at a laptop during a session; account of contact reduced to a blood-pressure measurement.	Institutional provision for attentive presence during the acute phase (Clinician-patient interaction; Normative pathway; recommendation R2).
Active physical grounding	Touch, particularly hand-holding, described by some participants as an external reference during ego dissolution. Raised by a minority.	Descriptions of hand-holding as an anchor to external reality.	Availability on conditions agreed in advance, with patient initiative retained (Normative pathway; recommendation R6).
Post-acute transition, “landing aid”	Absence of a marked ending described as abrupt discharge; a marked ending described as valuable. Reported in both contexts.	Being “spat out” or “discharged by the clock”; the counter-example of a shared cup of tea after the session.	A marked, structured closure as a routine component; specific form left open (Clinician-patient interaction; Normative pathway; recommendation R3).
Modes of experiencing safety	Three interpretive patterns: informed skeptic; relationship-oriented truster; autonomous self-regulator. Most participants combine features of more than one.	Use of neurobiological vocabulary to frame the experience; reliance on medical authority and warmth; self-regulation with eye mask and music.	Individualization above a non-negotiable clinical-safety floor, with the institution eliciting preferences (Modes of experiencing safety; Normative pathway; recommendation R4).
Models of mechanism of action	Three types: catalyst of transformative processes; moments of clarity and relief; pharmacological symptom management.	Descriptions of ketamine as opening a window for change; as temporary relief; as a medication whose subjective effects are side effects.	Eliciting the patient’s model to anticipate divergent expectations of preparation and integration (Models of mechanism; Normative pathway; recommendation R5).

## Discussion

This study examined patients’ and clinicians’ perspectives on safety in (es)ketamine treatment through qualitative content analysis of semi-structured interviews, in three domains: pre-treatment concerns, the role of clinician–patient interaction, and the influence of differing models of the mechanism of action on contextual requirements. Our findings suggest that safety in ketamine treatment is not a uniform phenomenon but a relationally created, individually differentiated and context-dependent experience emerging from the interplay of patient dispositions, clinician behaviors and environmental conditions. The following four subsections discuss each domain against previous research and, applying the framework set out in (Normative approach), identify the layer of vulnerability at issue; the section (From findings to recommendations) draws the findings together into the inferential pathway behind each recommendation.

### Pre-treatment concerns: beyond the “last resort” narrative

Previous research has identified that many patients approach ketamine as a “last resort” after prolonged treatment-resistant depression [20, 22, 23]. Our findings are consistent with this and extend it by identifying a differentiated typology of pre-treatment concerns that persists alongside the motivational pull of desperation: acute-effect worry, the framing of psychoactive effects as side effects, and an absence of worry.

These concerns arise within an information environment that is itself ambivalent. Whereas parts of the research literature frame the psychoactive effects as potentially meaningful, psychedelic-type experiences, the product information and regulatory framing present them as transient dissociative adverse effects to be monitored and tolerated. Patients who seek information therefore encounter conflicting accounts of what they are about to undergo, which plausibly contributes to the uncertainty expressed in both acute-effect worry and side-effect reframing.

Against this background, the distinction between patients who acknowledge their fear of LSD-like effects and those who manage it by reframing the psychoactive dimension as a mere side effect is instructive. Both groups appear anxious; what differs is how the anxiety is handled: named and tolerated in the first case, regulated defensively in the second by minimizing the significance of the experience so as to preserve a sense of control, a reframing that mirrors the product information and may therefore feel officially endorsed. While this may facilitate treatment entry, a patient who downplays the experiential dimension in advance may be poorly prepared for its intensity. This bears on informed consent in a specific and demanding sense: consent grounded in information the patient has received but not emotionally engaged with may be procedurally valid yet substantively thin. It deepens Stockwell et al.‘s observation [13] that a prepared mindset supports benefit: preparedness depends not on receiving information but on how patients engage with it.

The subgroup reporting no worries should likewise not be read as psychologically ready. Its absence of worry arose through routes distinct from preparedness: active avoidance of information to forestall anticipatory anxiety, which by design leaves patients less prepared; reassurance drawn from institutional signals such as insurance coverage, taken as a proxy for safety; and a dispositional pragmatism that does not entail that the experiential reality of the treatment has been considered. In depression, moreover, a resigned affect or the sense of having nothing left to lose may suppress the expression of worry without indicating readiness. This challenges he assumption that more information invariably yields better-informed decisions, since some patients actively decline it, but equally cautions against reading calm or compliance as readiness. 

In the terms of the section (Normative approach), these patterns locate where the induced layer of vulnerability is first triggered. The patient who reframes or avoids is, at the moment of consent, exercising the self-protective agency that the acute state will shortly reduce; preparation is therefore the last point at which the capacity to anticipate, ask and decline is unimpaired, and the treating party controls when that point passes. Because the trigger is scheduled and foreseeable, the absence of preparation addressed to the experience as such cannot be excused as unforeseeable, and the obligation it generates is not discharged by the patient’s own reluctance to hear more. Engaging agency rather than replacing it [36] here means offering preparation in a form the patient can accept, including the option of less detail, rather than withholding it or imposing it. This is the reasoning behind recommendation R1 (Table 2).

Finally, the finding that most pre-treatment worries did not intensify during treatment but gave way to a diffuse preoccupation with pre-existing anxieties suggests that the acute phase may be less dominated by treatment-specific fears than anticipated. This is consistent with Breeksema et al.‘s observation [21] that the capacity to “let go” is shaped by factors beyond the immediate treatment context.

### Clinician-patient interaction: from general relevance to specific mechanisms

A central contribution of this study is its analysis of how specific clinician behaviors shape felt safety during ketamine-induced altered states of consciousness. Previous research has established the general importance of therapeutic alliance, trust and clinician presence [10, 21–23]; the behavioral specifics through which safety is facilitated or disrupted have remained underexplored.

Our findings distinguish physical attendance from therapeutic presence. Clinicians engaging in administrative tasks such as using laptops during sessions were experienced as a profound rupture, described in terms of abandonment, dehumanization and industrial processing, which moves the discussion from a general call for presence to concrete behavioral specifications. The finding resonates with the literature on the relational dimensions of care [42, 52]. In the terms of our framework it marks the point at which clinical safety and felt safety come apart: in the laptop account, physiological monitoring proceeded correctly while the patient experienced the session as abandonment. The monitoring discharged the clinical-safety obligation; it did not discharge what is owed to a person whose capacity for self-advocacy has been reduced by the party now attending to them, and it is this second obligation that attentive presence answers (see From findings to recommendations; recommendation R2).

The identification of active physical grounding, particularly hand-holding, as an “external reference” during ego dissolution specifies the set-and-setting concept [14] at the level of embodied interaction: patients experience the clinician’s body as an anchor to external reality during profound internal disorientation, a finding that invites engagement with phenomenological accounts of embodiment and intersubjectivity in therapeutic contexts.

This finding requires ethical qualification and should not be read as establishing that physical contact is appropriate in general: it was raised by a minority of participants, and our material does not show whether others found it unwelcome, were not offered it, or did not think of it in interview. The question is not whether contact is permissible as such. A review of the arguments for and against physical touch in psychedelic-assisted treatment finds no author defending a categorical prohibition and reports a shift away from that framing toward asking which form of touch, under which conditions, is defensible [53]. It distinguishes safety touch, used to prevent physical harm and widely held to be permissible without prior explicit consent; supportive touch such as hand-holding, treated as comparatively uncontroversial where consent and risk-mitigation procedures are in place; and active touch or bodywork, which carries distinct risks and requires specialized training. What our participants describe is supportive touch, and our material bears on neither of the other two forms. That review concerns classical psychedelics and MDMA rather than (es)ketamine, but its own argument against psychedelic exceptionalism, that the questions raised by touch recur across nursing, psychotherapy and palliative care, tells against treating our setting as a special case; what differs is the degree of induced incapacity, which strengthens rather than relaxes the conditions below. Five considerations bear on whether, and under what conditions, supportive contact can be offered; their practical specification is given in Table 3, recommendation R6.

First, consent to contact must be sought before administration, while the patient is unimpaired, and must cover its form, location, duration and ending; but because assent may change during the acute phase and the capacity to communicate a change is precisely what the intervention reduces, a non-verbal stopping signal must be agreed in advance, ambiguity treated as withdrawal of assent, and the default under uncertainty must be non-contact, since the patient least able to object is the one for whom the safeguard matters most. Second, what is agreed in advance governs not only whether contact may be offered but how a request made during the session is to be handled, and the two directions are not symmetrical. The literature identifies the patient who asks for contact during the session having declined it in preparation as its least settled question, the disagreement turning on whether a decision taken in an induced state can count as authorization; it converges, so far as it converges at all, on the recommendation that the response to such a request be settled in advance rather than improvised in the moment [53]. Our analysis gives that agreement a determinate shape. Where a patient has said in advance that contact would be welcome, an approach or request initiated by the patient during the session may be followed, since the authorization was given while the capacity to give it was intact and the initiative remains the patient’s; the one account of hand-holding in our material was of exactly this form, contact asked for by the patient rather than offered by the clinician. Where a patient has declined contact in advance, a request made during the session should not be treated as reinstating it: the clinician should acknowledge the request, offer the non-contact alternatives agreed in preparation, and say that the preference can be revisited once the acute phase has passed. The asymmetry does not impute insincerity to the patient. It follows from who controls the trigger: the advance refusal was made in the condition in which the patient could weigh it, the in-session request in the condition the treating party induced, and treating the second as overriding the first would let the intervention dissolve the one protection the patient was in a position to put in place. Where contact was neither agreed nor declined because the question was not put, the default is non-contact, and the omission is a failure of preparation rather than a licence to improvise. Third, trauma histories are common in treatment-resistant depression, and our material contains an account of earlier trauma from prior treatment resurfacing during administration; for such a patient, touch initiated by a figure of clinical authority during a state of reduced capacity to refuse may reproduce the structure of the original experience, and because norms governing touch are culturally variable and gendered, the composition of the clinical team relative to the patient is material. Fourth, boundary norms governing physical contact in psychiatric care matter more, not less, during a consciousness-altering treatment, so availability should be governed by explicit institutional policy, with documentation, staff training and a route for patients to raise concerns afterwards, which protects clinicians as well as patients. Fifth, the non-contact alternatives described in our material, verbal orientation, the sustained presence of a clinician nearby, a held object, agreed music, or in one account a trusted companion, should be the starting point rather than the fallback: where grounding is what is needed, it does not follow that touch is what provides it. Where contact has been agreed, indirect forms such as pressure applied through a cushion, or contact from a trusted companion rather than from the clinician, have been proposed as ways of supplying the anchor while reducing the asymmetry that makes clinician-initiated contact problematic [53].

The concept of “landing aid”, developed from our data for the structured transition from the acute experience to everyday life, addresses a gap in the ketamine literature. Integration has been noted in the psychedelic therapy literature [54, 55] and mentioned for ketamine [10, 13]; our findings highlight that the immediate post-acute transition, not only long-term integration, is a critical and underrecognized phase, and accounts of being “spat out” or “discharged by the clock” suggest that current practice frequently fails to provide it. In the terms of the section (Normative approach), the unmarked ending is the clearest instance in our material of a cascading layer: the patient is left to reintegrate alone at the point of least capacity, and the resulting distrust and reluctance to voice needs propagate into later sessions without individual wrongdoing. As a theoretical extrapolation rather than a finding of this study, comparable requirements may arise in treatment with classical psychedelics, whose effects are more intense and more variable in duration, once such substances leave the resource-rich environment of clinical trials; our data concern (es)ketamine only. The normative weight of the finding is conditional: if institutions offering ketamine treatment have an obligation to minimize foreseeable harm, structured landing aid should be a standard component of care rather than an optional addition. The antecedent is defended, and its strength graded, in the section (From findings to recommendations).

### Modes of experiencing safety: individualized care as ethical imperative

The three modes of experiencing safety, the informed skeptic, the relationship-oriented truster and the autonomous self-regulator, describe how patients differentially construct safety in consciousness-altering treatment. Previous studies have noted individual variation in responses to the treatment context [20, 21]; no prior study has described such patterns for ketamine treatment.

Descriptively, these patterns suggest that a one-size-fits-all setting is unlikely to meet all patients’ needs: the informed skeptic, who derives safety from transparent, non-hierarchical communication and is sensitive to disruptions in the alliance, requires a different approach from the relationship-oriented truster, for whom medical authority and warmth provide a secure base, or from the autonomous self-regulator, for whom clinician interaction during the acute phase may be intrusive.

Normatively, the divergent cases reported in the Results (Clinician-patient interaction) are what the layered account predicts rather than anomalies. Layers of vulnerability are relations between a person and a circumstance, not properties of a population [37], so the same response, continuous presence, relieves a layer for one patient and constitutes an intrusion, possibly a new layer, for another; the participant who found interaction intrusive and the participant who needed the clinician beside them were both describing what made the induced state inhabitable for them. Two things follow. Because the relevant layers differ between patients, a uniform protocol will protect some and expose others, so individualization is an obligation rather than a courtesy. And because articulating needs is itself harder for those most affected, and our material shows patients suppressing needs they feared were burdensome, the duty to identify preferences falls on the institution, to be discharged by offering options proactively before administration, rather than on the patient to request them in a state of reduced capacity. This is the agency-engaging response Rogers and colleagues require [36], applied to the acute situation, and it is bounded as set out in (From findings to recommendations): individualization operates above the clinical-safety floor and never against it. The argument extends the relational analysis into a context in which dependency is pharmacologically induced rather than given.

### Models of mechanism and their treatment implications

The finding that patients’ and clinicians’ understandings of how ketamine works relate to their expectations of the treatment context is a further contribution. The three types, catalyst of transformative processes, moments of clarity and relief, and pharmacological symptom management, show that the implicit mechanism model functions as a framing device shaping what participants consider relevant, necessary and sufficient in preparation, accompaniment and integration.

A clinically and ethically significant pattern concerns the relation between mechanism model and engagement with care: Type 1 participants articulated more differentiated expectations of preparation and post-session processing than Type 3 participants, who more often described enduring discomfort without raising it. Three qualifications precede interpretation. The difference is partly definitional, since ascribed patient agency is one of the five dimensions from which the types were constructed. Clinicians are concentrated in Type 1 and are professionally fluent in experiential vocabulary, so the difference may track occupational role rather than mechanism model. And articulating fewer experiential requirements is not a deficit: a patient who seeks a pharmacological intervention without experiential accompaniment holds a coherent position, and our account of the modes of experiencing safety treats a preference for minimal interaction as legitimate. We therefore do not characterize participants holding a biomedical model as passive or insufficiently engaged. What our data support is a narrower, interactional claim: where a patient does not frame needs in experiential terms and the clinician does not enquire in those terms, an unmet need has no route into the encounter. The risk is misalignment, with needs unexpressed on one side and unrecognized on the other; we emphasize its possibility rather than its prevalence, since our sampling permits no inference about the relative frequency of the types (see Limitations).

Two implications follow: preparation may benefit from extending beyond psychoeducation toward a collaborative exploration of expectations and support needs, since what counted as relevant preparation varied with participants’ model of the treatment; and explicitly eliciting patients’ perspectives may help identify misalignment. In the terms of the section (Normative approach), the misalignment is a trigger the institution controls: the clinician’s model sets the default frame of the encounter, and a need that cannot enter it in the patient’s own terms is a need the induced state will leave the patient unable to press. Eliciting the model is thus the institutional duty to identify preferences proactively that the preceding subsection (Modes of experiencing safety), applied to expectations rather than atmosphere. We put the effect forward as a hypothesis: our design permits no inference to outcomes such as strengthened alliance or reduced discontinuation, which prospective research should test.

The tension between surrender and control identified across types deepens Breeksema et al.‘s findings [10, 21] on “letting go”: our analysis shows this capacity to be embedded in and enabled by the relational and contextual conditions of treatment rather than a trait, and surrender to be understood by patients as an active process requiring trust and support, with implications for how preparation and accompaniment are approached.

That caring clinician behavior and small gestures of personal concern can substantially mitigate structural and resource limitations is noteworthy from a practical-ethics perspective: even under constrained resources, relational quality, the experience of being seen and treated as a person, can buffer the effects of suboptimal environmental conditions.

## Recommendations

### From findings to recommendations: the normative pathway

This section sets out the reasoning by which the findings support the recommendations given in Recommendations for clinical practice, applying the framework of the section (Normative approach). Table 2 sets out one inferential pathway for each recommendation, labelled R1 to R6; the recommendations in Table 3 carry the same labels, so that every recommendation can be read against the finding, the ethical consideration and the reasoning from which it derives.

Patients entering (es)ketamine treatment carry layers of vulnerability that precede the treatment: the inherent vulnerability of embodiment and dependency, and the situational vulnerability of treatment-resistant depression, which involves both diminished agency and a constrained decision situation, since by definition the alternatives have been exhausted. In the IV infusion context a further economic layer arises from self-payment (see The two treatment contexts). None of these layers is created by the clinician.

The decisive layer, however, is created by the intervention. These treatments work by producing an acute state in which the patient’s capacity to monitor, evaluate, articulate and refuse is pharmacologically reduced. In Luna’s terms this layer is dispositional and its stimulus condition is the administration of the substance [38]. What distinguishes this case from most in which vulnerability is invoked in bioethics is that the trigger is scheduled, foreseeable and under the control of the treating party; the exposure cannot be described as unanticipated.

Two consequences follow, and they pull in different directions. First, causal history is morally relevant: the party to whom the patient becomes vulnerable is the party that induces the vulnerability, which, on the premises set out in (Normative approach), lifts the resulting obligations above a general duty of beneficence. Second, the induced layer is not simply to be minimized. On the therapeutic rationale of these treatments the relinquishment of control is part of what the treatment is, and several participants described it as valuable rather than merely endured. Following the argument that certain forms of vulnerability are conditions of agency rather than defects to be removed [56], we take the institutional obligation to be not to reduce the induced vulnerability but to render it inhabitable.

This is where the distinction drawn in (Goal and contribution of the present study) does analytic work. Clinical safety is discharged by monitoring, which is what the regulatory frame requires [16, 26]. Felt safety concerns whether the induced state can be inhabited. Ethical protection concerns what is owed given that the state was induced by those now responsible for the patient. The laptop account in the Results (Clinician-patient interaction) is a case in which the first was maintained and the other two were not.

Such failures do not remain local. Several accounts in our material have the structure of a cascading layer [36, 38]: an unmarked ending or an inattentive accompaniment is followed by reluctance to voice needs in later sessions, which in turn renders subsequent unmet needs less visible to clinicians. No wrongdoing on any individual’s part is required for this sequence, and identifying it as a cascading layer rather than as a failure of individual clinicians is both more accurate and more useful for institutional design.

A vulnerability-based analysis carries a characteristic risk: that responses to vulnerability entrench the passivity they describe, modelling care as a relation between a needy patient and a competent provider [56] and sliding into unwarranted paternalism [57]. We therefore treat it as a constraint on every recommendation that it must engage rather than replace patient agency. This constraint does substantive work below.

Consistent with the section (Normative approach), vulnerability contributes discrimination rather than a further premise: it identifies which layer is at issue, when and by whom it is triggered, and which responses relieve it and which propagate it. Table 2 sets out, for each recommendation in (Recommendations for clinical practice), the empirical finding, the ethical consideration invoked, the reasoning connecting them, the considerations that count against, and the resulting strength.

**Table 2:** Inferential pathways from empirical findings to recommendations. Each pathway (R1–R6) supports the recommendation carrying the same label in Table 3

No.	Empirical finding	Ethical consideration	Bridging reasoning	Competing considerations	Strength of normative support
R1	Pre-treatment uncertainty about what will happen and how far effects extend; information avoidance as anxiety management (Concerns before treatment; Information patients would have liked to receive)	Induced situational vulnerability with a foreseeable, clinician-controlled trigger [36, 37]	Where a susceptibility is deliberately actualized at a scheduled moment by the treating party, absence of preparation cannot be excused as unforeseeable. Preparation is the minimal form of respect owed to a person about to be placed in a state of reduced self-advocacy.	Excessive anticipatory information may itself generate anxiety and shape the experience through expectation; mandated materials already cover adverse effects, though not the experience as such [25].	Strong
R2	Therapeutic presence contrasted with administrative or procedural distance, reported in both treatment contexts (Results, Clinician-patient interaction)	Causal responsibility for induced dependency [36]; obligation to render the induced state inhabitable rather than merely survivable [56]	The party that induces the dependency is the party to whom the patient is then vulnerable. Discharging that responsibility requires attentive presence and not only monitoring, since monitoring addresses clinical safety while the patient’s need concerns felt safety.	Staffing and reimbursement constraints; some patients prefer minimal interaction; continuous presence can itself intrude.	Strong as a requirement on institutions to make attentive presence available; moderate as a requirement in every session
R3	Absence of structured transition; being “spat out”; the counter-example of shared closure, reported in both treatment contexts (Results, Clinician-patient interaction)	Cascading vulnerability [36, 38]; the regulatory frame defines session end as a discharge decision [16, 26]	An unmarked transition from an induced altered state to unsupervised everyday life leaves the patient to reintegrate alone at the point of least capacity. Where this fails, the resulting distrust and reluctance to voice needs constitute a further layer that propagates into subsequent sessions.	Resource cost; some patients report no such need; the boundary between transitional support and (psycho)therapeutic intervention requires competence and role clarity.	Strong for a marked, structured closure; weak for any specific form it should take
R4	Heterogeneity in what makes patients feel safe; three overlapping modes (Results, Clinician-patient interaction)	Layers rather than labels [37]; obligation to engage agency rather than entrench passivity [36, 56, 57]	Because the relevant layers differ between patients and are actualized by different triggers, a uniform protocol will protect some and expose others. Because articulating needs is itself harder for those most affected, the duty falls on the institution to offer options proactively rather than on the patient to request them.	Standardization serves clinical safety, comparability and equity; individualization can become a burden shifted onto the patient; unlimited accommodation is not feasible.	Moderate, and explicitly bounded: individualization operates above the clinical-safety floor, never against it
R5	Patients and clinicians hold divergent models of how ketamine works, and these are associated with divergent expectations of preparation, accompaniment and post-session processing (Models of mechanism of action)	Institutional control over the frame of the encounter; obligation to engage agency by identifying needs proactively rather than awaiting their articulation [36, 37]	The clinician’s model sets the default vocabulary of the encounter, so a need the patient cannot frame in that vocabulary has no route into it. Because the induced state removes the capacity to press a need that has gone unrecognized, the burden of eliciting the model falls on the party that controls the frame, and must be discharged before the state is induced	The association between model and articulated need is partly definitional and may track occupational role rather than model; eliciting models costs consultation time and imposes a framing that some patients may not want	Moderate; supported as a communicative requirement, not as a demonstrated effect on outcomes
R6	Active physical grounding, including hand-holding, reported as stabilizing during ego dissolution (Results, Clinician-patient interaction)	Anti-paternalism constraint [56, 57]; risk of converting a protective response into a cascading layer [38]	Touch offered during an induced state cannot be meaningfully declined at the moment of offering, and would be initiated by the party responsible for the reduced capacity to decline. A supportive act under these conditions reproduces the asymmetry it is meant to relieve unless authorization was secured while the patient was unimpaired and the patient retains initiative. Advance permission and advance refusal are not symmetrical: permission plus patient initiative supplies what is needed, whereas an in-session request cannot reinstate a permission the patient declined while unimpaired, since the capacity to weigh it is what the intervention removes.	The reported benefit is substantial and the finding robust; over-formalization may remove a resource patients value; declining all touch is not neutral and may be experienced as withdrawal; no author in the reviewed literature defends a categorical prohibition, and some hold that discounting a preference expressed during the session is itself countertherapeutic [53].	Weak as our findings alone would suggest it; revised and conditional as formulated in Recommendations for clinical practice

Strength of normative support refers to the strength of the normative argument developed in this empirical-ethical analysis, that is, how well the empirical findings and the ethical considerations together support each recommendation. It does not express the certainty of clinical evidence and does not follow GRADE or any other formal guideline-grading system. All recommendations are proposals arising from an exploratory qualitative study.

Applying this normative framework, that is, the vulnerability analysis introduced in (Normative approach) and worked through row by row in Table 2, changes what we recommend relative to what the empirical findings alone would support: it has altered one recommendation, physical grounding (R6), and bounded another, individualization (R4). Our findings on physical grounding, taken alone, would support recommending that clinicians offer contact such as hand-holding during the acute phase. The analysis does not permit this: touch offered during an induced state cannot be meaningfully declined at the moment of offering and would be initiated by the party responsible for the reduced capacity to decline, so that a supportive act under these conditions reproduces the asymmetry it is meant to relieve and, where unwelcome, is a candidate for the cascading effect described above. We therefore recommend that physical grounding be available only on conditions agreed while the patient is unimpaired, with the patient retaining initiative, and that advance permission and advance refusal be treated asymmetrically: the first, together with patient initiative, licenses contact during the session, while the second is not reinstated by a request made in the induced state (see Recommendations for clinical practice). We record this explicitly because the revision runs against our own finding: the reports of benefit are genuine, and it is the framework rather than the data that generates the restriction. Our finding of heterogeneity likewise supports individualization, which the anti-paternalism constraint and the felt/clinical safety distinction bound as set out in the Discussion (Modes of experiencing safety).

### Recommendations for clinical practice

The recommendations below follow from the analysis in From findings to recommendations, where the empirical finding, the ethical consideration invoked, the reasoning connecting them and the considerations against are set out for each (Table 2); the labels R1 to R6 identify the corresponding pathway in that table. They are stated here with the strength of their normative support and their conditions, and should be read with the Limitations. None of them rests on demonstrated effects on clinical outcomes or clinical trials, which our design cannot establish.

**Table 3:** Recommendations for clinical practice, with strength of normative support and conditions. The labels R1–R6 refer to the corresponding inferential pathways in Table 2

Recommendation	Strength of normative support	Conditions and qualifications
R1. Individualize pre-treatment preparation, addressing not only factual knowledge but patients’ implicit models of how the treatment works, their coping strategies for anticipated effects, and their preferred level of information.	Strong	Attend particularly to patients who minimize or avoid information, which may indicate anxiety management rather than disinterest. Excessive anticipatory detail can itself raise anxiety and shape the experience through expectation.
R2. Make attentive presence available during the acute phase, and address the conduct of accompanying staff explicitly in clinical protocols.	Strong for institutional provision; moderate as a requirement in every session	Administrative activity during sessions was described as destabilizing even where clinicians regarded it as neutral. Some patients prefer minimal interaction; presence should be available rather than imposed. Staffing and reimbursement constraints are real and are not addressed by this recommendation.
R3. Provide a marked, structured transition out of the acute phase (“landing aid”), with an adequate time buffer before departure.	Strong for a marked closure; weak for any specific form	Our data do not establish which form of closure is preferable; brief debriefing, ritualized transition and unhurried time were all described. The boundary between transitional support and (psycho)therapeutic intervention requires competence and role clarity.
R4. Elicit individual safety preferences before treatment and accommodate them in the atmospheric, relational and temporal features of the session.	Moderate, and explicitly bounded	Individualization operates above a non-negotiable baseline of clinical safety and monitoring; where preference conflicts with a monitoring requirement, the requirement prevails. Because difficulty in articulating needs is itself part of the vulnerability at issue, the duty to identify preferences falls on the institution and is discharged by offering options proactively.
R5. Explore patients’ and clinicians’ models of how ketamine works, in order to anticipate divergent expectations of preparation, accompaniment and integration.	Moderate	Our design permits no inference to outcomes such as strengthened alliance or reduced discontinuation; the recommendation rests on the risk of unrecognized misalignment, not on demonstrated effects.
R6. Settle the question of supportive physical contact such as hand-holding before administration. Where the patient has agreed to it, follow the patient’s own initiative during the session. Where the patient has declined it, do not offer it in response to a request made during the session, and do not offer it on the clinician’s own assessment of the patient’s state. Safety touch necessary to prevent physical harm is not governed by this recommendation.	Conditional; not recommended as a routine component of care	Agree form, extent, ending and a non-verbal stopping signal in advance, and agree at the same time how a request made during the session is to be handled. Advance permission and advance refusal are not symmetrical: permission licenses following the patient’s own initiative, whereas refusal is not reinstated by an in-session request, which should be met with the agreed non-contact alternatives and the offer to revisit the preference afterwards. Treat ambiguity during the session as withdrawal of assent; default to non-contact, and treat an unasked question in preparation as a failure of preparation rather than a licence to improvise. Include trauma history in preparation; take cultural norms and the gender composition of the team into account. Govern availability by explicit institutional policy with documentation, training and a route for later concerns. Provide non-contact alternatives as the starting point, including indirect contact or contact from a companion where these would serve [53]. This restriction follows from our vulnerability analysis (see From findings to recommendations) and runs against our own empirical finding, which reported physical grounding as stabilizing in our sample.

Strength of normative support is used as defined in the note to Table 2: it grades the normative argument developed in this analysis, not the certainty of clinical evidence, and follows no formal guideline-grading system. All recommendations are proposals arising from an exploratory qualitative study.

### Limitations

Several limitations should be considered in interpreting these findings and the recommendations derived from them in the Recommendations section.

First, convenience sampling may have introduced selection bias: patients who agreed to participate may differ from those who declined, for instance by being more reflective about their experiences, more positively or more negatively inclined toward the treatment, or more comfortable with self-disclosure. Transferability to broader patient populations and clinical contexts should therefore be assessed with caution.

Related to this, the contextual description of our sample is limited by the data-minimization decision reported in (Participant selection and setting). Three consequences follow. Readers cannot establish how far our participants resemble their own patients, so judgements of transferability must rest on the treatment contexts described in (The two treatment contexts). We cannot exclude that features of the safety experience we report are associated with characteristics we did not record, such as stage of treatment, cumulative exposure or prior experience with psychoactive substances; in particular, accounts given during and after a course of treatment may differ systematically, and our judgement that the point in the course was not decisive for our questions is defensible but was not tested. And because clinicians cannot be matched to the patients they treated, no inference runs from an individual clinician’s account to an individual patient’s experience. These are real costs of the data-minimization decision; future studies should collect aggregated contextual descriptors, which can be done without compromising anonymity, and record stage of treatment at interview.

Further, we do not claim thematic saturation, and the concerns, safety modes and mechanism models we report should not be read as exhaustive. Further interviews might have yielded additional themes or variants, particularly in the two subgroups in which our material is thinnest, clinicians and patients treated by infusion. The modes and types organize the variation present in this sample and are not a complete account of how safety may be experienced in these treatments.

Second, and most consequentially, treatment modality is confounded with the model of accompanying care in our sample. All infusion patients received some form of integration work; the esketamine context includes no such component, either at the participating sites or in the regulatory and manufacturer framework, which defines the end of a session as a checklist-supported discharge decision [16, 26]. The confound is a property of the participating sites rather than of the modalities, since integration work is not standard in German intravenous ketamine treatment, where the practice literature describes a monitored rest phase [47] and treats ketamine-augmented psychotherapy as investigational [43]. Nor is it the only difference: the contexts also differ in route and invasiveness, in patient control over intake, in regulatory status and in funding, since esketamine is covered by statutory insurance whereas our infusion patients paid for treatment themselves [44, 46], which is not incidental given that one patient described insurance coverage as reassuring and others described reluctance to voice needs they perceived as burdensome.

Our design cannot separate these influences, and the infusion group, drawn from several sites without a common protocol, is not internally homogeneous either. We therefore make no comparative claims between the modalities and do not interpret differences in the distribution of themes or types as modality effects. Our findings should be read as an account of what shapes felt safety in supervised treatment involving acute alterations of consciousness, with the caveat that the relative weight of the requirements identified may differ across settings that differ in invasiveness, patient control, funding and intensity of accompanying care. Future studies should sample both modalities under comparable models of care.

Third, the study was conducted in the German healthcare context, with insurance-funded access to intranasal esketamine, a particular regulatory environment and specific cultural attitudes toward mental health treatment and psychoactive substances. Whether the findings, particularly regarding institutional trust and insurance approval as a source of reassurance, transfer to systems with different structures remains an open question.

Fourth, no methods of enhanced trustworthiness such as member checking, peer debriefing or independent audit of the coding were employed; the interpretive validity of the findings rests on the transparency and plausibility of the analytical process as documented here.

Fifth, interviews were conducted mainly in German, with excerpts translated into English for publication. Despite efforts to preserve nuance and to give original terms in brackets, some semantic loss is inevitable, most consequentially for the affectively loaded metaphors that made expressions analytically significant, such as being “spat out” (ausgespuckt), the “rooster in the yard” (Hahn im Korb) or an “external reference” (Referenz im Außen).

Finally, qualitative content analysis, while suited to descriptively rich and practically oriented findings, does not aim at theory building in the manner of grounded theory or at the experiential depth of interpretative phenomenological analysis. The modes and types are empirically grounded interpretive frameworks rather than theoretical models; their value lies in informing practice and generating hypotheses for further investigation.

## Conclusions

This study examined how patients and clinicians experience safety in supervised (es)ketamine treatment, and what follows ethically from those experiences.

Patients’ pre-treatment concerns proved more differentiated than the “last resort” narrative suggests: alongside desperation for an effective treatment, participants described fears about acute psychoactive effects, reframed those effects as side effects, or avoided information in order to regulate anxiety. Consent grounded in information a patient has not engaged with emotionally may be procedurally valid yet substantively thin.

Felt safety was described as relationally constituted and is not secured by physiological monitoring alone. Participants in both treatment contexts distinguished attentive presence from administrative distance, and described the unmarked end of a session as the point at which support was most needed and least available. Because the diminished capacity for self-regulation and self-advocacy is induced by the intervention, and by those who then care for the patient, the quality of relational care carries obligations that a general duty of beneficence would not generate. We distinguish this throughout from clinical safety, which sets a floor that preference cannot lower, and from ethical protection, which neither of the other two establishes on its own.

Safety was also not experienced uniformly. The modes of experiencing safety and the types of mechanism model describe patterns in what participants needed and in how they understood the treatment, and suggest that a uniform protocol will suit some patients and expose others. We present them as provisional interpretive patterns rather than validated profiles.

What these findings identify are features of practice rather than of the pharmacology of the substance: how information is given, how clinicians conduct themselves during the acute phase, how a session is closed, and how individual differences are elicited. Our data cannot establish what changing them would cost or achieve. As an exploratory qualitative study in one national context, with a small sample and without a systematic assessment of saturation, these findings require examination in larger and more diverse samples, sampling both modalities under comparable models of accompanying care and testing prospectively whether the requirements identified here affect treatment experience, adherence or outcome.

## Supplementary Information

Supplementary Material 1.

Supplementary Material 2.

Supplementary Material 3.

Supplementary Material 4.

Supplementary Material 5.

Supplementary Material 6.

Supplementary Material 7.

Supplementary Material 8.

Supplementary Material 9.

Supplementary Material 10.

## Data Availability

The qualitative data analysed during this study are not publicly available in order to protect participant privacy, as the comprehensive nature of the transcripts carries a high risk of deductive disclosure. On reasonable request, the corresponding author can provide access to selected, de-identified portions of the data. Codebooks and Interview Guidelines are available in the preregistration: https://osf.io/94rtw.

## Abbreviations

COREQ: Consolidated Criteria for Reporting Qualitative Research
E: Expert/clinician (participant code)
EMA: European Medicines Agency
FDA: Food and Drug Administration
IVI: Intravenous infusion (participant code)
NVL: Nationale VersorgungsLeitlinie (German National Care Guideline)
S: Spravato/esketamine nasal spray (participant code)
