# Supplementary material for: Feeling safe: an empirical-ethical analysis of vulnerability in (es)ketamine treatment
Source: BMC Med Ethics. 2026 Oct 8;27:218. doi: 10.1186/s12910-026-01637-7 (PMC13659964; doi:10.1186/s12910-026-01637-7)
Supplement: Supplementary file 1 [file 12910_2026_1637_MOESM10_ESM.docx]

**Additional file 10: Category system for the analysis of mechanism-of-action models and treatment setting**

*Feeling Safe: An Empirical-Ethical Analysis of Vulnerability in (Es)ketamine Treatment*

This file reproduces the category system developed for the analysis of participants' models of ketamine's mechanism of action and of the associated treatment setting. As reported in section 2.5 of the manuscript, the 20-category system documented in Additional file 6 proved insufficiently aligned with this research question, and a separate system of six main categories was developed inductively for this component. This constitutes a departure from the preregistration and is reported as such.

One main category captures models of the mechanism of action. It is differentiated into the five dimensions that constitute the feature space of the typology reported in section 3.3; each dimension carries three nested values corresponding to the typological differentiation. The remaining five main categories organize setting-related material, each subdivided according to whether a statement concerned the perceived relevance of a feature (subcategory R) or its implementation in practice as experienced or described by the participant (subcategory P). A residual category was retained for statements not assignable to these five.

**Table A. Model of mechanism of action: feature space of the typology**

| **Dimension** | **Nested values (typological differentiation)** | **Definition** |
| --- | --- | --- |
| Mechanistic Understanding | Neuroplastic Model; Vague or Hybrid Explanations; Mechanism Irrelevant or Unknown | Accounts of how ketamine exerts its antidepressant effects |
| Clinical Role and Value | Transformative Opportunity; Ambivalent Positioning; Last-Resort Option | Perceptions of ketamine's position within the broader spectrum of depression treatments |
| Experiential Significance | Transformative or Insightful; Moderately Meaningful or Ambivalent; Experiential Side Effect | Ascribed value to the subjective experience under ketamine |
| Efficacy Assessment | Process Focus; Acute Relief; Symptom Focus | Conceptualization of ketamine's effect |
| Patient Agency | Active Engagement; Partial Engagement; Passive Reception | The degree to which the patient is considered active in the therapeutic process |

The five dimensions above are the dimensions across which cases were characterized in the type-building analysis. Cases were grouped polythetically, by overall similarity across the dimensions rather than by any single shared criterion; no case matches its type on every dimension. Case-level values are given in Additional file 9.

**Table B. Treatment setting: main categories and nested subcategories**

| **Main category** | **Nested subcategories in subcategory R (relevance)** | **Nested subcategories in subcategory P (practice)** |
| --- | --- | --- |
| Preparation | Building Trust; Clarifying Expectations | General Psychoeducation; Individualized Consultation |
| Treatment Environment | Comfort and Safety; Calmness and Stability; Immersion and Grounding | Room Design; Institutional Environment; Augmentative Elements |
| Clinician Presence | Safety; Orientation; Connection | General Presence; Verbal Interaction; Physical Contact |
| Post-Treatment Transition | Rest and Recovery; Reassurance and Connection | Time Pressure and Overstimulation; Routines and Structure |
| Post-Session Processing | Preparation and Focusing; Understanding and Anchoring; Exchange and Processing | Clarification or None; Debriefing and Integration; Self-Directed Integration |
| Additional Relevant Aspects | Clinical Setting; Group Setting; General Statements | None |

**Table C. Distribution of codings across the six main categories, by type**

The following three tables report the distribution of coded segments across the categories of this system for all 24 participants entering the type-building analysis, that is 10 clinicians and 14 patients. Counts are given per type rather than per case, since the analytic unit of that component is the type. They are reported as context for the qualitative analysis and do not constitute a quantitative weighting in a statistical sense. The corresponding distribution for the eleven categories analysed in the study of safety experience, which rests on patient interviews only, is given in Additional file 8.

| **Main category** | **Type 1** | **Type 2** | **Type 3** | **Total** |
| --- | --- | --- | --- | --- |
| Clinician Presence | 46 | 49 | 20 | 115 |
| Treatment Environment | 49 | 22 | 34 | 105 |
| Post-Session Processing | 50 | 18 | 13 | 81 |
| Preparation | 42 | 14 | 8 | 64 |
| Post-Treatment Transition | 14 | 4 | 5 | 23 |
| Additional Relevant Aspects | 14 | 5 | 8 | 27 |
| **Total** | **215** | **112** | **88** | **415** |
| **N = documents** | **8 (33.3%)** | **8 (33.3%)** | **8 (33.3%)** | **24 (100%)** |

**Table D. Distribution of codings across the dimensions of the mechanism-of-action feature space, by type**

| **Dimension and nested value** | **Type 1** | **Type 2** | **Type 3** | **Total** |
| --- | --- | --- | --- | --- |
| ***Patient Agency*** | | | | |
| Passive Reception | – | – | 1 | 1 |
| Partial Engagement | 8 | 4 | 3 | 15 |
| Active Engagement | 9 | – | – | 9 |
| ***Efficacy Assessment*** | | | | |
| Symptom Focus | – | 13 | 11 | 24 |
| Acute Relief | 7 | 12 | 1 | 20 |
| Process Focus | 20 | 1 | – | 21 |
| ***Experiential Significance*** | | | | |
| Experiential Side Effect | 1 | 3 | 14 | 18 |
| Moderately Meaningful or Ambivalent | 4 | 18 | 7 | 29 |
| Transformative or Insightful | 29 | 3 | 1 | 33 |
| ***Clinical Role and Value*** | | | | |
| Last-Resort Option | 1 | 5 | 28 | 34 |
| Ambivalent Positioning | 11 | 13 | 8 | 32 |
| Transformative Opportunity | 6 | 6 | – | 12 |
| ***Mechanistic Understanding*** | | | | |
| Mechanism Irrelevant or Unknown | – | 5 | 4 | 9 |
| Vague or Hybrid Explanations | 7 | 7 | 6 | 20 |
| Neuroplastic Model | 12 | 4 | – | 16 |
| **Total** | **115** | **94** | **84** | **293** |
| **N = documents** | **8 (33.3%)** | **8 (33.3%)** | **8 (33.3%)** | **24 (100%)** |

The gradient across the three types is visible in this distribution. Type 1 accounts for all codings of Active Engagement and for the large majority of Process Focus, Transformative or Insightful, and Neuroplastic Model; Type 3 accounts for all codings of Passive Reception and for the large majority of Last-Resort Option and Experiential Side Effect. We note that the dimension of ascribed patient agency is one of the criteria from which the types were constructed, so that a difference along it partly restates a classification criterion rather than constituting an independent finding; this is discussed in section 4.4 of the manuscript.

**Table E. Distribution of codings across the setting subcategories, by type**

Within each of the five setting categories, subcategory R records statements concerning the perceived relevance of a feature and subcategory P statements concerning its implementation in practice, as described above.

| **Category and subcategory** | **Type 1** | **Type 2** | **Type 3** | **Total** |
| --- | --- | --- | --- | --- |
| ***Additional Relevant Aspects*** | | | | |
| General Statements | 4 | 2 | 1 | 7 |
| Group Setting | 5 | 1 | – | 6 |
| Clinical Setting | 5 | 2 | 7 | 14 |
| ***Post-Treatment Transition – P (practice)*** | | | | |
| Routines and Structure | 1 | 1 | 2 | 4 |
| Time Pressure and Overstimulation | 7 | – | – | 7 |
| ***Post-Treatment Transition – R (relevance)*** | | | | |
| Reassurance and Connection | 3 | 2 | 2 | 7 |
| Rest and Recovery | 3 | 1 | 1 | 5 |
| ***Preparation – P (practice)*** | | | | |
| Individualized Consultation | 6 | 2 | – | 8 |
| General Psychoeducation | 12 | 6 | 6 | 24 |
| ***Preparation – R (relevance)*** | | | | |
| Clarifying Expectations | 11 | 1 | 2 | 14 |
| Building Trust | 13 | 5 | – | 18 |
| ***Post-Session Processing – P (practice)*** | | | | |
| Self-Directed Integration | 5 | – | – | 5 |
| Debriefing and Integration | 7 | 3 | 1 | 11 |
| Clarification or None | 12 | 11 | 11 | 34 |
| ***Post-Session Processing – R (relevance)*** | | | | |
| Exchange and Processing | 6 | – | – | 6 |
| Understanding and Anchoring | 13 | 3 | – | 16 |
| Preparation and Focusing | 7 | 1 | 1 | 9 |
| ***Treatment Environment – P (practice)*** | | | | |
| Augmentative Elements | 4 | 11 | 6 | 21 |
| Institutional Environment | 8 | – | 5 | 13 |
| Room Design | 11 | 4 | 6 | 21 |
| ***Treatment Environment – R (relevance)*** | | | | |
| Immersion and Grounding | 4 | 4 | 4 | 12 |
| Calmness and Stability | 10 | 2 | 7 | 19 |
| Comfort and Safety | 12 | 1 | 6 | 19 |
| ***Clinician Presence – P (practice)*** | | | | |
| Physical Contact | 6 | 6 | 1 | 13 |
| Verbal Interaction | 10 | 12 | 3 | 25 |
| General Presence | 9 | 16 | 14 | 39 |
| ***Clinician Presence – R (relevance)*** | | | | |
| Connection | 3 | 4 | – | 7 |
| Orientation | 5 | 1 | – | 6 |
| Safety | 13 | 10 | 2 | 25 |
| **Total** | **215** | **112** | **88** | **415** |
| **N = documents** | **8 (33.3%)** | **8 (33.3%)** | **8 (33.3%)** | **24 (100%)** |

Two observations from this distribution are taken up in the manuscript. Physical contact is among the least frequently coded subcategories of clinician presence (13 codings across 24 participants), which is the basis for the statement in section 4.2 that active physical grounding was raised by a minority of participants. And within post-session processing, the subcategory Clarification or None is the most frequently coded across all three types (34 codings), which corresponds to the finding reported in section 3.2 concerning the absence of a structured post-acute transition.
