# Supplementary material for: Feeling safe: an empirical-ethical analysis of vulnerability in (es)ketamine treatment
Source: BMC Med Ethics. 2026 Oct 8;27:218. doi: 10.1186/s12910-026-01637-7 (PMC13659964; doi:10.1186/s12910-026-01637-7)
Supplement: Supplementary file 2 [file 12910_2026_1637_MOESM1_ESM.docx]

**Additional file 1: COREQ (COnsolidated criteria for REporting Qualitative research) checklist**

Manuscript: Feeling Safe: An Empirical-Ethical Analysis of Vulnerability in (Es)ketamine Treatment.

*Page numbers refer to the revised manuscript. Where an item was not applicable or the information was not collected, this is stated rather than left blank.*

| **No.** | **Item** | **Guide question / description** | **Reported in (section, page)** |
| --- | --- | --- | --- |
| **Domain 1: Research team and reflexivity** | | | |
| ***Personal characteristics*** | | | |
| 1 | Interviewer / facilitator | Which author/s conducted the interview or focus group? | Patient interviews by AK, JG or HS; clinician interviews by AK. Section 2.1, p. 9 |
| 2 | Credentials | What were the researcher's credentials? | AK: PhD, qualifications in clinical psychology, psychotherapy and philosophy. JG, HS: MSc psychology students at the time of the study. Section 2.1, p. 9 |
| 3 | Occupation | What was their occupation at the time of the study? | AK: postdoctoral researcher, part-time research post at a German medical university. JG, HS: psychology students completing research internships and master's theses. Section 2.1, p. 9 |
| 4 | Gender | Was the researcher male or female? | AK male; JG and HS female. Section 2.1, p. 9 |
| 5 | Experience and training | What experience or training did the researcher have? | All interviewers had previous experience in qualitative clinical interviewing. Section 2.1, p. 9 |
| ***Relationship with participants*** | | | |
| 6 | Relationship established | Was a relationship established prior to study commencement? | No prior personal or professional relationship with any interviewed patient; some clinicians were known to one researcher from professional contexts. No researcher held clinical responsibility for any participant. Sections 2.1, p. 9; 2.3, p. 13 |
| 7 | Participant knowledge of the interviewer | What did the participants know about the researcher and the research goals? | Written study information plus verbal explanation of the purpose of the study and the interviewer's interest, with opportunity to ask questions before consent. Section 2.1, p. 9 |
| 8 | Interviewer characteristics | What characteristics were reported about the interviewer? Bias, assumptions, motivations, interests. | Team composition, disciplinary backgrounds, stance toward the treatment (agnostic to sceptical), absence of commercial or institutional stake, and the sequencing of researchers across data collection and interpretation are reported. Section 2.1, pp. 9–10 |
| **Domain 2: Study design** | | | |
| ***Theoretical framework*** | | | |
| 9 | Methodological orientation and theory | What methodological orientation was stated to underpin the study? | Structuring qualitative content analysis with type-building analysis (Kuckartz; Kuckartz and Rädiker); normative approach stated separately as empirical bioethics with a layered-vulnerability framework. Sections 2.2, pp. 10–11; 2.2.1, pp. 11–12 |
| ***Participant selection*** | | | |
| 10 | Sampling | How were participants selected? | Convenience sampling with a snowball component; patients from cooperating clinical sites, clinicians from these institutions or professional networks. Section 2.3, p. 13 |
| 11 | Method of approach | How were participants approached? | Patients informed by clinicians at cooperating sites who passed on the research team's contact details; interviews then arranged by the team. Voluntariness safeguards reported. Section 2.3, p. 13; Additional file 2, section A |
| 12 | Sample size | How many participants were in the study? | 24 participants: 14 patients (9 esketamine, 5 infusion) and 10 clinicians. No participant was excluded and no case was omitted from a component for want of codeable material; the part of the corpus addressed by each component is specified in Section 2.5. Sections 2.3, pp. 13–14; 2.5, pp. 18–21 |
| 13 | Non-participation | How many people refused to participate or dropped out? Reasons? | Not systematically recorded, because sites forwarded only the details of patients willing to be interviewed; stated explicitly with reasons. Section 2.3, p. 13 |
| ***Setting*** | | | |
| 14 | Setting of data collection | Where were the data collected? | Patient interviews in person or online according to preference; clinician interviews online. Treatment settings described separately. Sections 2.3, p. 14; 2.3.1, pp. 15–16, and Additional file 3 |
| 15 | Presence of non-participants | Was anyone else present besides the participants and researchers? | Interviews were conducted one to one with no one else present. Section 2.3, p. 14 |
| 16 | Description of sample | What are the important characteristics of the sample? | Modality, indication, sex, age range (27–66 years), treatment stage at interview, clinician professional groups and the two treatment contexts; items not collected are stated explicitly. Sections 2.3, pp. 14–15; 2.3.1, pp. 15–16, and Additional file 3 |
| ***Data collection*** | | | |
| 17 | Interview guide | Were questions, prompts, guides provided by the authors? Was it pilot tested? | Semi-structured guide developed for the study, piloted by the German and Taiwanese teams (one pilot interview each); reproduced in Additional file 4 and published in the preregistration. Section 2.4, p. 17 |
| 18 | Repeat interviews | Were repeat interviews carried out? | No repeat interviews were conducted with the same participant for the present analysis. Section 2.4, p. 17 |
| 19 | Audio / visual recording | Did the research use audio or visual recording to collect the data? | Audio-recorded using dedicated recording devices. Section 2.4, p. 17 |
| 20 | Field notes | Were field notes made during and/or after the interview or focus group? | No field notes were made during or after interviews. Section 2.4, p. 17 |
| 21 | Duration | What was the duration of the interviews or focus group? | Clinician interviews 24–62 minutes (mean 38); patient interviews 23–134 minutes (mean 42). Section 2.4, p. 17 |
| 22 | Data saturation | Was data saturation discussed? | Discussed and not claimed. No systematic saturation assessment was carried out; the claim made is one of information power. Section 2.4, p. 17; Additional file 2, section B; Limitations, p. 56 |
| 23 | Transcripts returned | Were transcripts returned to participants for comment and/or correction? | No; participants did not provide feedback on transcripts or findings. Section 2.4, p. 18 |
| **Domain 3: Analysis and findings** | | | |
| ***Data analysis*** | | | |
| 24 | Number of data coders | How many data coders coded the data? | Three coders (AK, HS, JG), calibrated in three rounds on one complete study transcript each (3 of 24 transcripts) until mean pairwise percentage agreement across all codes reached 80% (MAXQDA Intercoder Agreement, segment level); each analytic component then coded by a single researcher (AK, JG, HS respectively). Section 2.5, pp. 19–20 |
| 25 | Description of the coding tree | Did authors provide a description of the coding tree? | Development from 623 codes to 20 main categories described; the 11 categories selected for differentiation are named; full code system, codebook and category distributions provided as Additional files 5–8. Section 2.5, pp. 19–21 |
| 26 | Derivation of themes | Were themes identified in advance or derived from the data? | Deductive–inductive: main categories specified in advance, 36 subcategories derived inductively from the material; modes of experiencing safety and mechanism-of-action types derived from the material; all 14 patient interviews are coded at main- and subcategory level, and all 24 participants entered the type-building analysis. Section 2.5, pp. 18–23 |
| 27 | Software | What software, if applicable, was used to manage the data? | MAXQDA version 24.11.1. Section 2.5, p. 18 |
| 28 | Participant checking | Did participants provide feedback on the findings? | No. Stated explicitly, together with the basis on which trustworthiness rests. Section 2.5, p. 22 |
| ***Reporting*** | | | |
| 29 | Quotations presented | Were participant quotations presented to illustrate the themes / findings? Was each quotation identified? | Yes; every quotation is identified by an anonymized participant code, with the coding convention stated at the start of the Results. Sections 2.5, p. 22; 3, p. 24 |
| 30 | Data and findings consistent | Was there consistency between the data presented and the findings? | Themes, modes, types, illustrative evidence and the implications drawn are set out together in Table 1; divergent and negative cases are reported. Section 3.2, p. 32; Table 1, p. 37 |
| 31 | Clarity of major themes | Were major themes clearly presented in the findings? | Presented by section with illustrative quotations and summarized in Table 1. Section 3, pp. 24–37 |
| 32 | Clarity of minor themes | Is there a description of diverse cases or discussion of minor themes? | A dedicated passage reports divergent and negative cases, including counter-examples to the main patterns. Section 3.2, p. 32 |

Note on data handling and use of language models. Large language models were not used in data collection, transcription, coding, category development, typology construction, the derivation of the modes of experiencing safety or the selection of quotations. No identifiable participant data were entered into any model at any stage, nor were any research data, that is, transcripts, audio recordings, coding files or the MAXQDA project; the dataset is held on secured local servers of Charité – Universitätsmedizin Berlin. In preparing the revised manuscript and the additional files the authors used a large language model (Claude, Anthropic) for language editing and for drafting and restructuring passages of text. The material entered into the model consisted solely of manuscript text, including anonymized quotations already selected by the authors for publication; the responsible data protection officer confirmed that this does not constitute processing of personal data. All analytic decisions, normative positions and the final text are the authors' own, who take full responsibility for the manuscript. The model is not an author. See Section 2.6, p. 23.

Reference: Tong A, Sainsbury P, Craig J. Consolidated criteria for reporting qualitative research (COREQ): a 32-item checklist for interviews and focus groups. Int J Qual Health Care. 2007;19(6):349–57.
