# Supplementary material for: Feeling safe: an empirical-ethical analysis of vulnerability in (es)ketamine treatment
Source: BMC Med Ethics. 2026 Oct 8;27:218. doi: 10.1186/s12910-026-01637-7 (PMC13659964; doi:10.1186/s12910-026-01637-7)
Supplement: Supplementary file 3 [file 12910_2026_1637_MOESM2_ESM.docx]

**Additional file 3: The two treatment contexts**

*Feeling Safe: An Empirical-Ethical Analysis of Vulnerability in (Es)ketamine Treatment*

This file gives the full description of the two treatment contexts from which participants were drawn, summarized in section 2.3.1 of the manuscript. It sets out the regulatory framework, the mode and schedule of administration, the monitoring requirements and the model of accompanying care for each, and closes with a comparison across the dimensions on which they differ.

**1. Intranasal esketamine (Spravato®; participants coded S)**

Esketamine nasal spray has been licensed in Germany since March 2021 for treatment-resistant depression and, in a second indication, for the rapid reduction of depressive symptoms in situations judged by a physician to constitute a psychiatric emergency. In Europe it is approved only in combination with an oral antidepressant, not as monotherapy.

The National Care Guideline for unipolar depression gives it an open recommendation for moderate to severe episodes that have not responded to several adequate treatment attempts, formulated for the partial or full inpatient setting; a passage restricting its availability to such settings was removed in version 3.2 of the guideline in July 2023, following a change in the terms of its authorization. Since the Federal Joint Committee recognized an additional benefit in September 2023, statutory health insurers are obliged to cover the treatment. The patients in our sample received esketamine as outpatients at hospital outpatient clinics.

The prescribing decision must be made by a psychiatrist. The product is not dispensed to the patient: the pharmacy supplies it to the treating practice or clinic, where the patient self-administers it under the direct supervision of clinical staff. The patient therefore retains a degree of physical control over intake that has no counterpart in infusion treatment, operating the device and using one, two or three single-use applicators at five-minute intervals for doses of 28, 56 or 84 mg, resting in a comfortable, preferably semi-reclined position after each. Food is avoided for two hours and drink for thirty minutes beforehand. The standard schedule is eight sessions across four weeks, followed by weekly and then optionally fortnightly maintenance, so that patients attend the same setting repeatedly over months.

A treatment session comprises the administration and a subsequent period of post-administration observation, both of which must take place in an appropriate medical setting. Monitoring is defined in physiological terms: blood pressure is measured before administration, again at approximately forty minutes, and thereafter at clinical discretion; observation continues until staff judge the patient stable enough to be discharged. The European framework sets no fixed duration, and the length of the observation period at the participating sites accordingly varied with individual patients' reactions within the parameters set by the product information. The manufacturer's guidance for healthcare professionals, distributed in Germany as risk-minimization material under the terms of the marketing authorization, instructs clinicians to make the discharge judgement using a checklist and reports that most patients in one study reached that point within ninety minutes. Patients are advised not to drive or perform other demanding activities until the following day.

In the German setting, initial administration typically takes place under the supervision of a physician, with subsequent monitoring by nursing staff and a physician available on call. We did not systematically record the staffing arrangements at the individual sites, and patients' accounts of who was present and how are analyzed as part of the findings rather than treated as a fixed feature of the setting.

Two features of this framework bear directly on the questions of this study. First, the acute psychoactive effects are classified throughout as adverse reactions: dissociation is listed among the very common undesirable effects, reported in 27% of treated patients, alongside dizziness, nausea and somnolence. Second, neither the regulatory framework nor the educational materials mandated as a condition of the marketing authorization provide for psychological preparation for the acute experience or for its subsequent processing. As a condition of the authorization the marketing authorization holder must operate a controlled access programme in each member state and agree the content and format of the educational material with the national competent authority; that material comprises a guide for healthcare professionals, a checklist whose stated purpose is to help staff judge when a patient is stable enough to be discharged safely, and a patient guide covering adverse effects, the risk of abuse and dependence, preparation rules on food and drink, blood-pressure monitoring, the need for observation until judged clinically stable, and the effect on driving. Nothing in this mandated content concerns preparation for the acute experience as an experience, or its subsequent processing. The end of a treatment session is defined at regulatory level as a discharge decision supported by a stability checklist. The absence of structured integration in the esketamine context is therefore not an incidental feature of the participating sites but is built into the standard care model for this modality.

**2. Intravenous racemic ketamine infusion (participants coded IVI)**

Ketamine infusion for depression is off-label in Germany. The National Care Guideline issues a strong recommendation against its use outside an inpatient psychiatric setting, seeing an indication only in individual cases of the most severe treatment resistance and conditional on the treatment being embedded in an overall psychiatric concept; the guideline group explicitly names the risk of commercially motivated provision as a privately billed service. As an off-label treatment it is not routinely reimbursed by statutory health insurance, in contrast to esketamine, although the substance itself is many times cheaper per dose and is not scheduled under the German narcotics act. Notwithstanding the guideline, the treatment is also delivered in day-clinic and outpatient settings, and its tolerability there has been described favourably in the German literature. The patients in our sample received infusions as outpatients, within institutional protocols at several different sites, and paid for the treatment themselves.

The protocol described in the German practice literature is a subanaesthetic dose of 0.5 mg per kilogram of body weight, delivered by syringe driver over forty to forty-five minutes in a carrier solution rather than as a bolus, commonly as a series of six administrations at two per week, followed by individually determined maintenance. Sessions are to take place in a quiet, low-stimulus environment, with noise-cancelling headphones and a sleep mask recommended as means of shielding the patient from stimuli, explicitly as a way of managing possible psychotomimetic effects. Vital signs and clinical observation are recommended at intervals of five to ten minutes. The first administration takes place under anaesthesiological supervision or in the care of a physician experienced with ketamine, subsequent ones in the presence of a member of the nursing staff with the responsible physician within call. Antiemetic and antihypertensive medication is kept ready. The acute phase is followed by a rest and observation period of approximately one and a half to two hours with continued monitoring, after which patients are advised not to drive or operate machinery for twenty-four hours.

We report this protocol as the reference point described in the German practice literature, not as a documented account of the participating sites. Because ketamine infusion is off-label there is no binding standard protocol, practice varies locally, and we interviewed patients rather than institutions. What patients themselves reported about dose, setting, stimulus regulation, who was present and how they were accompanied is treated as data and analyzed in the Results rather than presupposed here. All patients in this group received some form of integration work alongside the infusions; its content, timing and intensity differed between individuals and sites.

It should be noted that integration work of this kind is not a standard element of intravenous ketamine treatment in Germany. The practice literature describes the post-infusion period as a monitored rest phase, and treats ketamine-augmented psychotherapy as an investigational approach whose central assumption, that ketamine-induced plasticity opens a therapeutic window, remains difficult to test empirically. The infusion patients in our sample therefore received more than the German norm, which is a property of the participating sites rather than of the modality.

**3. Comparison of the two contexts**

| **Dimension** | **Intranasal esketamine** | **Intravenous ketamine infusion** |
| --- | --- | --- |
| **Regulatory status** | Licensed for treatment-resistant depression; add-on to an oral antidepressant only | Off-label; guideline advises against use outside inpatient settings |
| **Funding** | Covered by statutory health insurance | Not routinely reimbursed; patients in this sample paid privately |
| **Administration** | Self-administered nasal spray, one to three applicators at five-minute intervals | Clinician-administered infusion by syringe driver, venous access required |
| **Patient control over intake** | Patient operates the device and can pause between applicators | None once the infusion has started |
| **Invasiveness** | Non-invasive | Invasive |
| **Duration of acute phase** | Short; effects typically subside within about 1.5 hours | Infusion over 40–45 minutes plus 1.5–2 hours of monitored rest |
| **Monitoring** | Blood pressure before administration and at ~40 minutes, then at clinical discretion; observation until judged stable | Vital signs and clinical observation every 5–10 minutes throughout |
| **End of session** | Discharge decision supported by a stability checklist | End of the monitored rest period |
| **Frequency** | 8 sessions over 4 weeks, then weekly to fortnightly maintenance | Commonly 6 administrations at 2 per week, then individual maintenance |
| **Accompanying care** | No preparation or integration component in the regulatory or manufacturer framework | Structured integration work at the participating sites; not standard for the modality in Germany |
| **Internal homogeneity** | Single licensed framework | Several sites, no common protocol |

*The sources on which this description rests are cited in section 2.3.1 of the manuscript and listed in its reference list: the European and German product information and the associated risk-minimization materials for esketamine; the German National Care Guideline for unipolar depression; and the German practice and review literature on subanaesthetic ketamine infusion.*
