# Supplementary material for: Feeling safe: an empirical-ethical analysis of vulnerability in (es)ketamine treatment
Source: BMC Med Ethics. 2026 Oct 8;27:218. doi: 10.1186/s12910-026-01637-7 (PMC13659964; doi:10.1186/s12910-026-01637-7)
Supplement: Supplementary file 4 [file 12910_2026_1637_MOESM3_ESM.docx]

**Additional file 2: Supplementary methodological notes**

*Feeling Safe: An Empirical-Ethical Analysis of Vulnerability in (Es)ketamine Treatment*

This file sets out in full three methodological matters that are summarized in the manuscript: the safeguards for voluntariness and the procedures in case of distress during recruitment and interviewing (section A, summarized in section 2.3 of the manuscript); the basis of the preregistered sample-size target and the reasoning behind the claim of information power rather than saturation (section B, summarized in section 2.4); and the provisions under which the mechanism-of-action types and the modes of experiencing safety are reported (section C, summarized in section 2.5). Reference numbers refer to the reference list of the manuscript.

**A. Recruitment ethics: voluntariness and procedures in case of distress**

Patients were informed about the study by clinicians at the cooperating sites, who were project partners in recruitment and who passed on the research team's contact details; patients then approached the team, or agreed to be contacted by it. Patients were informed that participation was voluntary and that neither participation nor refusal would have any positive or negative consequence for their treatment. Refusals were not communicated to the cooperating clinicians by the research team, and the team was therefore not in a position to make a patient's non-participation known. We cannot exclude that individual patients disclosed their decision to their clinicians themselves, which was outside the research team's control and of which we have no knowledge. Because sites forwarded only the details of patients willing to be interviewed, participation was by definition visible to the informing clinician, while refusal was not. No researcher held any clinical responsibility for any interviewed patient, and no member of the research team was involved in the care of any participant. No compensation was offered for participation.

Procedures were in place in case an interview caused distress. At the start of each interview participants were told verbally that they could decline any question, could pause or end the interview at any point without giving a reason, and could contact the interviewer afterwards if distress became apparent only later. At the end of each interview participants were asked how they were and whether they were experiencing distress or wished for any form of support. No interview had to be interrupted on account of distress, and no participant required support after an interview. Participants were informed that they could withdraw their participation and have their data deleted, in accordance with applicable German law.

**B. Sample size, saturation and information power**

The target sample size was specified in advance in the preregistration, which set a target of 17 interviews per participant group on the basis of a systematic review of empirical tests reporting that saturation in comparable studies is typically reached between nine and 17 interviews [48]. This is an expectation drawn from the general literature rather than a determination about the present study, and we do not claim on that basis that thematic saturation was achieved. No systematic assessment of saturation was carried out. We did not apply a formal stopping criterion of the kind operationalized by Francis and colleagues [50], such as a specified number of consecutive interviews yielding no new codes, and we did not document the emergence of new codes across successive interviews in a way that would allow diminishing novelty to be demonstrated rather than asserted. Adequacy was not assessed separately for patients and clinicians, for the two treatment modalities, or for the three analytic components, and no team member carried out a systematic assessment of diminishing novelty. Data collection was governed by access to willing participants rather than by an analytic stopping rule, and the preregistered target of 17 per group was not reached in either group.

We therefore make the more cautious claim that the sample provides sufficient information power for the aims of this study [49], rather than that saturation was achieved. Four considerations support this. The aims are exploratory and descriptive rather than confirmatory. The study population is narrow, comprising patients undergoing one class of treatment for one indication together with clinicians delivering it, which increases the information yield per case. The interviews were conducted with a topic-specific guide and produced dense material, with the safety-relevant categories alone yielding extensive coded content across the corpus (Additional file 8). And the analysis applies established methodological frameworks rather than seeking to generate new theory, which lowers the sample size required. Information power is weakest for the clinician sample and for the ketamine-infusion group, the smaller of the two treatment groups. The distinction between code saturation (the point at which no new codes are formed) and meaning saturation (the broader state in which the content of each code is fully understood) [51] is relevant here: even if the former is plausible for our central categories, we make no claim to the latter. The consequences for how the types and modes should be read are stated in the Limitations of the manuscript.

**C. Provisions under which the types and modes are reported**

For the type-building analysis of mechanism-of-action models, types were constructed polythetically within the five-dimensional feature space documented in Additional file 10, so that cases were grouped by overall similarity across the dimensions rather than by any single shared criterion; no case matches its type on every dimension, and the types are consequently internally heterogeneous. Cases that combined features of more than one type were assigned on the balance of dimensions and, where this remained unclear, on the account of the mechanism of action itself, that dimension being the one the typology is about. The initial classification was carried out by the researcher who coded this component (HS) and was subsequently reviewed against the coded material with the first author (AK); divergent judgements were resolved by returning to the case summaries, and cases that could not be characterized across the dimensions would have been left unassigned rather than allocated to the nearest type; in the event all 24 cases could be characterized and none was left unassigned. The resulting case-to-type relationships and the distribution of patients, clinicians and treatment modalities across the types are given in Additional file 9.

Because assignments rest on interpretive judgements about whole cases, we report the types as provisional interpretive patterns rather than as validated classifications, and the type labels are shorthand for those patterns rather than clinical profiles or characterizations of the individuals concerned. The modes of experiencing safety reported in section 3.2.1 of the manuscript are subject to the same provisions and carry them more strongly: they are overlapping interpretive patterns derived from case summaries rather than the result of formal type construction against a predefined feature space, cases were not exclusively assigned to one mode, most participants combined features of more than one, and no case-to-mode assignment is therefore reported (Additional file 9, section C).
