# Supplementary material for: Feeling safe: an empirical-ethical analysis of vulnerability in (es)ketamine treatment
Source: BMC Med Ethics. 2026 Oct 8;27:218. doi: 10.1186/s12910-026-01637-7 (PMC13659964; doi:10.1186/s12910-026-01637-7)
Supplement: Supplementary file 5 [file 12910_2026_1637_MOESM4_ESM.docx]

**Additional file 4: Semi-structured interview guide**

*Feeling Safe: An Empirical-Ethical Analysis of Vulnerability in (Es)ketamine Treatment*

This file reproduces the semi-structured interview guide used in the present study, as developed within the PsyTrans project and applied in the German sub-study. The guide was piloted by the German and Taiwanese teams before use. Questions are given in English and in the German wording actually used in the interviews.

The guide is a semi-structured instrument: the questions below were used as a flexible frame rather than as a fixed sequence, and were adapted to what each participant had already experienced at the time of the interview and to the course the conversation took. Not every question was put to every participant, and follow-up questions were asked where an answer invited them. The guide also contains sections for a non-clinical community sample (recreational users and retreat guides), which were not used for the analyses reported in this manuscript and are therefore not reproduced here.

**Questions for patients**

| **No.** | **English** | **German (as used)** |
| --- | --- | --- |
| 1 | How did you know about this treatment option / this study? | Wie haben Sie von dieser Behandlungsmethode / Studie erfahren? |
| 2 | What is your understanding of what psychedelics do? | Was ist Ihr Verständnis davon, wie Psychedelika wirken? |
| 3 | Where does this understanding come from? | Woher gelangen Sie zu diesem Verständnis? |
| 4 | Why did you decide to try this treatment option / participate in the study? | Warum haben Sie sich entschieden, diese Behandlungsmethode zu versuchen / an dieser Studie teilzunehmen? |
| 5 | What are your expectations regarding the treatment process? | Was sind Ihre Erwartungen an den Behandlungsverlauf? |
| 6 | Can you explain where these expectations come from? | Können Sie erklären, woher diese Erwartungen stammen? |
| 7 | Assuming the treatment runs as you expected, how do you imagine the role of the clinical professionals (e.g. therapist) in this treatment? | Angenommen die Behandlung verläuft wie von Ihnen erwartet, wie würden Sie sich die Rolle Ihres/Ihrer BehandlerIn (z.B. TherapeutIn) in diesem Prozess vorstellen? |
| 8 | Why do you imagine the role as you did? | Warum stellen Sie sich diese Rolle so vor? |
| 9 | Can you describe the course and your perspective on the medical issues that led you to try this treatment option / participate in the study? | Können Sie den Verlauf Ihrer gesundheitlichen Beschwerden beschreiben, der Sie in diesen Behandlungsversuch / in diese Studie geführt hat, und wie Sie persönlich darauf blicken? |
| 10 | Do you have prior experience using or being treated with psychedelic substances, and can you describe them? | Haben Sie Vorerfahrungen in der Nutzung oder Behandlung mit psychedelischen Substanzen, und können Sie diese beschreiben? |
| 11 | If you have had such experiences, how relevant are they to your current treatment / participation in the study? | Wenn Sie solche Erfahrungen gemacht haben, wie relevant sind diese für Ihre gegenwärtige Behandlung / Studienteilnahme? |
| 12 | Do you have any concerns about this treatment / participating in this study? (e.g. personal, ethical, financial and medical worries or anxieties) | Haben Sie irgendwelche Bedenken bezüglich der Behandlung / Ihrer Teilnahme an der Studie? (z.B. Sorgen, Ängste, persönliche, finanzielle oder medizinische Bedenken) |
| 13 | What is the attitude toward psychedelic substances in your family, among your friends, or in the society you live in? How do these attitudes influence you? | Was ist die Haltung in Ihrer Familie, unter Ihren Freunden und in der Gesellschaft, in der Sie leben, gegenüber psychedelischen Substanzen? Wie haben Sie diese Einstellungen beeinflusst? |
| 14 | Could you describe your experience during the acute effect of the substance? | Bitte beschreiben Sie Ihre Erfahrungen unter dem akuten Einfluss der Substanz. |
| 15 | Could you describe your interactions with others during the treatment process? | Können Sie beschreiben, wie Sie mit anderen interagiert haben während der Behandlung? |
| 16 | Could you describe your feelings about the environment where the treatment took place? | Können Sie beschreiben, wie Ihre Gefühle gegenüber der Umgebung waren, in der Sie behandelt wurden? |
| 17 | Did you think about any treatment expectations during the acute effect? | Haben Sie über Ihre Erwartungen an die Behandlung nachgedacht, während Sie unter dem akuten Einfluss der Substanz standen? |
| 18 | During the treatment, were there any concerns that occupied your mind? (e.g. worries, anxieties, or lingering thoughts) | Gab es während der Behandlung irgendwelche Bedenken, die Sie beschäftigt haben? (z.B. Sorgen, Ängste oder wiederkehrende Gedanken) |
| 19 | Have you experienced any changes after the treatment? (e.g. symptoms, psychological changes, beliefs, worldview, insights, life style, spiritual connection) | Haben Sie irgendwelche Veränderungen nach der Behandlung erlebt? (z.B. Symptome, psychologische Überzeugungen, Weltbild, Einsichten, Lebensstil, Spiritualität) |
| 20 | What role did the contact with your clinical professionals (e.g. therapists) before, during and after the treatment play for you? | Welche Rolle hat der Kontakt mit Ihren BehandlerInnen (z.B. TherapeutIn) vor, während und nach der Behandlung für Sie gespielt? |
| 21 | Were your expectations of the acute drug effects met? Which ones were not met? | Waren Ihre Erwartungen an die akute Auswirkung der Substanz zutreffend? Welche waren nicht zutreffend? |
| 22 | Were your expectations of the overall treatment met? Which ones were not met? | Waren Ihre Erwartungen an die Behandlung als Ganzes zutreffend? Welche waren nicht zutreffend? |
| 23 | Is there anything now after the treatment that you wish you had known beforehand? | Gibt es jetzt nach der Behandlung etwas, von dem Sie sich gewünscht hätten, es vorher zu wissen? |
| 24 | How does your financial situation shape your experience? | Wie hat Ihre finanzielle Situation Ihre Erfahrung beeinflusst? |

**Questions for clinicians**

| **No.** | **English** | **German (as used)** |
| --- | --- | --- |
| 1 | How did you first come into contact with the topic of psychedelic substances / ketamine or psychedelic treatment? | Wie sind Sie zum ersten Mal mit dem Thema psychedelische Substanzen / Ketamin oder der Behandlung mit Psychedelika in Kontakt gekommen? |
| 2 | What is your attitude towards these treatments? | Wie ist Ihre Einstellung zu diesen Behandlungen? |
| 3 | What is the attitude toward psychedelic substances in professional groups, family, friends, or the society you live in? How do these attitudes influence you? | Was ist die Einstellung zu psychedelischen Substanzen unter Ihren FachkollegInnen, in Ihrer Familie, Ihren Freunden und in der Gesellschaft, in der Sie leben? Wie haben Sie diese Einstellungen beeinflusst? |
| 4 | Please describe the treatment setting and the process of the treatment you offer. | Bitte beschreiben Sie das Behandlungsprozedere und das Behandlungssetting, das Sie anbieten. |
| 5 | Please describe the forms of interaction and your experience with patients (e.g. during the intervention / outside of the intervention). | Bitte beschreiben Sie, wie Sie mit PatientInnen interagieren und die Erfahrungen, die Sie dabei mit den PatientInnen machen (z.B. während der Intervention / abseits der Intervention). |
| 6 | How do you methodologically assess the changes in patients throughout the treatments? | Welche Methoden nutzen Sie, um die Veränderungen der PatientInnen über den Verlauf der ganzen Behandlung zu bestimmen? |
| 7 | What changes in patients do you observe? | Welche Veränderungen beobachten Sie bei PatientInnen? |
| 8 | How do you think, feel, evaluate, or conceptualize these observed changes in patients? | Was denken und fühlen Sie bezüglich der beobachteten Veränderungen? Wie evaluieren und konzeptualisieren Sie diese? |
| 9 | Which parts of your clinical training, values, and background knowledge play a particular role in your work in these treatments? | Welcher Teil Ihrer klinischen Ausbildung, welche Werte und welches Hintergrundwissen spielen eine besondere Rolle in Ihrer Arbeit mit diesen Behandlungen? |
| 10 | What types of people do you think are suitable or unsuitable to be treated with psychedelic substances? | Welche Personen sind in Ihren Augen geeignet oder ungeeignet für die Behandlung mit psychedelischen Substanzen? |
| 11 | What is your expectation of the development of psychedelic treatment / therapy? | Was ist Ihre Erwartung an die weiteren Entwicklungen im Feld der Behandlung mit psychedelischen Substanzen? |
