# Supplementary material for: Feeling safe: an empirical-ethical analysis of vulnerability in (es)ketamine treatment
Source: BMC Med Ethics. 2026 Oct 8;27:218. doi: 10.1186/s12910-026-01637-7 (PMC13659964; doi:10.1186/s12910-026-01637-7)
Supplement: Supplementary file 6 [file 12910_2026_1637_MOESM5_ESM.docx]

**Additional file 6: System of 20 main categories**

*Feeling Safe: An Empirical-Ethical Analysis of Vulnerability in (Es)ketamine Treatment*

This file reproduces the system of 20 main categories that resulted from the reduction of the initial 623-code system (Additional file 5). Reduction proceeded in a group consensus process on the criteria described in section 2.5 of the manuscript: codes were merged where their content overlapped, removed where they were rarely or never applied, and recalibrated so that each category corresponded to a meaningful unit of analysis. This system was applied to the entire interview corpus. The definitions reproduced below are the category memos held in the project codebook (version 1.4) and are given here in their original working formulation. The eleven categories selected for further inductive differentiation in the present analysis are marked with an asterisk; their subcategories are documented in Additional files 7 and 8.

| **No.** | **Category** | **Definition (project codebook memo)** |
| --- | --- | --- |
| 1 | Background knowledge | Background knowledge (or beliefs) the patients report that are not related to psychedelics. This may include medical expert knowledge, knowledge about psychopathology, and neuroscience. |
| 2 * | Patient Journey | This code is applied to narrative descriptions and their elements of patients' experiences up to the psychedelic treatment. Not for things that happened during the psychedelic treatment itself. This might also include knowledge by experience of their previous treatments and illness (e.g., how they react to certain psychotropic drugs). |
| 3 * | Knowledge of Psychedelic Treatment | Knowledge of psychedelic treatment is intended to code for the passages in which the patient expresses some ideas about how to understand how the psychedelic treatment works or should be carried out. "Knowledge" is not narrowly understood. If the patient has a spiritual model, or a psychological model, or a somehow inaccurate medical model of how psychedelics work, it is all considered their knowledge. |
| 4 * | Attitude toward Psychedelic Treatment | Attitude of patients towards different aspects of the treatment in general and towards the topic of psychedelic treatment. This is different from "evaluation" code, which is about the retrospective evaluation of experiences only, and it is different from "goals" code in that this code would be about a prospective perspective. |
| 5 * | Personal and Psychological Concerns of Psychedelic Treatment | Concerns (Ger: Sorgen, Bedenken) about how one might change psychologically or personally by undergoing the psychedelic treatment. |
| 6 | Social, Ethical, and Political Concerns of Psychedelic Treatment | Concerns (Ger: Sorgen, Bedenken) regarding legal consequences, social reactions, and ethical failure related to the decision to undergo psychedelic/ketamine treatment. |
| 7 | Spiritual, Religious, Philosophical, Metaphysical Concerns of Psychedelic Treatment | Concerns (Ger: Sorgen, Bedenken) regarding religious questions, the nature of the world and reality, of "what there is" and the "meaning of what there is". |
| 8 | Goals of Psychedelic Treatment | This code is intended to be used to code parts of the transcript in which the patients speak about what they want or wanted to achieve with the treatment. |
| 9 * | Roles of or Relationship with Healthcare and Medical Professionals | This code is intended to code for descriptions of interactions with healthcare professionals in context of psychedelic treatment or thoughts about the role of the relationship. It is not intended to code interactions with previous clinicians the patient encountered on their clinical journey. |
| 10 * | Roles of or Relationship with Non-Medical Individuals | Description of treatment, relevant personal relationships, and interactions with non-clinical individuals during treatment. |
| 11 * | Psychedelic Experiences | All psychological experiences that show a psychedelic quality (all senses etc.). |
| 12 * | Influences of Treatment Costs | Explicit mentioning of treatment cost for the patient as a factor in the decision for, experience of, and thoughts about the treatment. |
| 13 * | Influences of Treatment Environment | Treatment settings are described or discussed concurrently by the patient in relation to their experience in treatment. Not only mentioning different settings (there are hospitals and treatment modalities). |
| 14 | Influences of Cultural, Social, Political Environment | Socio-cultural influences (e.g., social media platforms, news, podcasts, blogs, etc.) on decision for/against treatment. In contrast to the influence of single social contacts and significant others. This code also includes remarks on features of the health systems in the country. |
| 15 | Intrapersonal Changes after Psychedelic Treatment | Lasting changes in experience (including side effects lasting longer than the psychedelic effects) and psychological changes in attitudes or behaviours. Not purely physiological changes (e.g., heart rate changes). |
| 16 | Interpersonal Changes after Psychedelic Treatment | Explicit mentioning that a relationship has changed in consequence of treatment (not only interpersonal change that leads to behavioural changes on the side of the patient). |
| 17 | Social, Ethical or Political Changes after Psychedelic Treatment | Changes of one's perspectives on social, ethical, or political issues |
| 18 | Spiritual, Religious, Philosophical, Metaphysical Changes after Psychedelic Treatment | Change of perspectives after treatment on religious questions, the nature of the world and reality, of "what there is" and the "meaning of what there is". |
| 19 * | Evaluation of Psychedelic Treatment | Retrospective evaluative remarks about the experiences with the substance, the setting, or the set. |
| 20 * | Suggestions for Psychedelic Treatment | Suggestions for alternative arrangement of treatment elements or procedures |

** Selected for further inductive differentiation in the analysis of safety experience reported in this manuscript.*
