# Supplementary material for: Feeling safe: an empirical-ethical analysis of vulnerability in (es)ketamine treatment
Source: BMC Med Ethics. 2026 Oct 8;27:218. doi: 10.1186/s12910-026-01637-7 (PMC13659964; doi:10.1186/s12910-026-01637-7)
Supplement: Supplementary file 7 [file 12910_2026_1637_MOESM6_ESM.docx]

**Additional file 5: Initial code system (623 codes)**

*Feeling Safe: An Empirical-Ethical Analysis of Vulnerability in (Es)ketamine Treatment*

This file reproduces the initial code system developed deductively within the PsyTrans project before analysis, from the overall project framework and the interview guide (Additional file 4). It was designed to cover the full thematic range of ketamine-based and psychedelic-assisted treatment across both national contexts of the project, which is why it is considerably broader than the analysis reported here requires. It comprises 11 parent codes and 612 subordinate codes, that is 623 in total. Codes were documented in English and Mandarin; only the English designations are reproduced here.

The codes are listed without definitions or anchor examples, which reflects the stage at which the system was used rather than a gap in its documentation. It was piloted in an exploratory, bootstrapping manner: the deductive codes were applied in a deliberately vague, common-sense reading, in order to establish which participant utterances they would in fact attract and what those utterances could contribute to defining them. Fixing definitions in advance would have foreclosed the question the piloting was meant to answer. The definitional work was therefore done in the second, consensus step, which reduced the system to the 20 main categories reproduced with their definitions in Additional file 6; the subcategories later developed within the eleven categories selected for the analysis of safety experience are documented in Additional file 7.

| **No.** | **Code** |
| --- | --- |
| **Knowledge of (39 codes)** | |
| 1 | recreational drugs |
| 2 | psychedelics |
| 3 | shaman |
| 4 | mental illness |
| 5 | addiction |
| 6 | substance abuse |
| 7 | religion |
| 8 | mystical experience |
| 9 | therapist training |
| 10 | IRB |
| 11 | informed consent |
| 12 | psychological preparedness |
| 13 | legal framwork |
| 14 | treatment protocol |
| 15 | risk management |
| 16 | support system |
| 17 | personal readiness |
| 18 | Pharmacology |
| 19 | Clinical Assessment |
| 20 | Medical Monitoring |
| 21 | Risk Stratification |
| 22 | Treatment Protocol Design |
| 23 | Evidence Base |
| 24 | Interdisciplinary Coordination |
| 25 | Documentation Requirements |
| 26 | treatment journey |
| 27 | set and setting |
| 28 | experience navigation |
| 29 | integration process |
| 30 | safety information |
| 31 | pracitcal logistics |
| 32 | Support Role Understanding |
| 33 | Safety Protocols |
| 34 | Integration Support |
| 35 | Practical Care Needs |
| 36 | Communication Guidelines |
| 37 | Personal Boundaries |
| 38 | Education Needs |
| 39 | Legal/Medical Context |
| **Expectation of X (31 codes)** | |
| 1 | Engnlsih |
| 2 | Symptoms improving |
| 3 | Risk of suicide |
| 4 | Disliked by anti-drug advocates |
| 5 | Mystical experiences |
| 6 | Recalling unpleasant memories |
| 7 | Self-exploration |
| 8 | Ego dissolution/Ego death state |
| 9 | Out-of-body experience |
| 10 | Experience of contact with supernatural beings |
| 11 | Transcending reality |
| 12 | Psychic abilities |
| 13 | Seeing the past |
| 14 | Hallucinations |
| 15 | Changes in body sensations |
| 16 | Emotional fluctuations |
| 17 | Dizziness |
| 18 | Vomiting |
| 19 | Feeling hot |
| 20 | Feeling cold |
| 21 | Trembling/Shaking |
| 22 | Drowsiness |
| 23 | Weakness in limbs |
| 24 | Rapid breathing |
| 25 | Spatial disorientation |
| 26 | Loss of balance |
| 27 | Seeing the future |
| 28 | Trauma healing |
| 29 | Delusions |
| 30 | Character change |
| 31 | Change is social relationships |
| **Concern of (22 codes)** | |
| 1 | Cost/Expense |
| 2 | Illegal |
| 3 | Addiction |
| 4 | Family opposition |
| 5 | Life-threatening danger |
| 6 | Going off to become a monk/nun |
| 7 | Isolating oneself from others |
| 8 | Being different from before |
| 9 | Negative comments from others |
| 10 | Suicidal thoughts |
| 11 | Becoming someone you don't like |
| 12 | Damaging health |
| 13 | Mental confusion/cloudiness |
| 14 | Abandoning family/children |
| 15 | Loss of self |
| 16 | Wanting to use again after using |
| 17 | Strong side effects |
| 18 | Being treated as an experiment subject |
| 19 | Worry about lack of effectiveness |
| 20 | Worry about too strong effects |
| 21 | Unpleasant experiences produced by the drug |
| 22 | Strange/judgmental looks from the public |
| **Attitude towards (109 codes)** | |
| 1 | psychedelics |
| 2 | drugs |
| 3 | elicit drus |
| 4 | therapeutic effects |
| 5 | risks |
| 6 | side effects |
| 7 | safety |
| 8 | scientific research |
| 9 | legalization |
| 10 | 政策 |
| 11 | social acceptance |
| 12 | spiritual or religious aspects |
| 13 | personal experience |
| 14 | past use expereince |
| 15 | the role of medical professionals |
| 16 | patient autonomy |
| 17 | support and care |
| 18 | traditional vs. emerging therapies |
| 19 | personal growth |
| 20 | transformation |
| 21 | clinical trials |
| 22 | cultural influence |
| 23 | dependency and misuse |
| 24 | death and end-of-life |
| 25 | cultural perception |
| 26 | reality and illusion |
| 27 | social stigma |
| 28 | time and space |
| 29 | collective healing |
| 30 | indigenous knowledge |
| 31 | media representation |
| 32 | altered states of knowledge |
| 33 | subjective vs. objective truth |
| 34 | epistemic authority |
| 35 | rationality vs. intuition |
| 36 | being and consciousness |
| 37 | mind-body relationship |
| 38 | agency and autonomy |
| 39 | embodiment |
| 40 | medical ethics |
| 41 | informed consent |
| 42 | accessibility and privilege |
| 43 | responsibility and harm reduction |
| 44 | psychedelic capitalism |
| 45 | indigenous rights |
| 46 | Study design and methodology |
| 47 | Blinding and expectancy effects |
| 48 | Mechanisms of action |
| 49 | Standardization of dosing |
| 50 | Long-term effects |
| 51 | Replication crisis and reproducibility |
| 52 | Participant selection criteria |
| 53 | Neuroimaging and biomarker validity |
| 54 | Interdisciplinary collaboration |
| 55 | Commercial influence on research |
| 56 | Therapist vs. non-therapist guides |
| 57 | Cultural bias in psychedelic research |
| 58 | Ethical use of placebo |
| 59 | Risk-benefit ratio |
| 60 | Psychedelics in healthy populations |
| 61 | AI and machine learning in psychedelic research |
| 62 | Legal classification and research limitations |
| 63 | Open science vs. proprietary data |
| 64 | Place of subjective reports in clinical studies |
| 65 | Harm reduction vs. abstinence approaches |
| 66 | Suitability of patients |
| 67 | Therapeutic frameworks |
| 68 | Pre-session preparation |
| 69 | Session setting and environment |
| 70 | Guided vs. self-directed experiences |
| 71 | Emotional intensity and catharsis |
| 72 | Touch and physical support |
| 73 | Use of complementary practices |
| 74 | Boundaries and ethical concerns |
| 75 | Risk of re-traumatization |
| 76 | Session frequency and duration |
| 77 | Post-session integration |
| 78 | Contraindications and medication interactions |
| 79 | Therapist self-experience with psychedelics |
| 80 | Regulation of psychedelic-assisted therapy |
| 81 | Cost and accessibility of therapy |
| 82 | Use of psychedelics outside clinical settings |
| 83 | Role of medical oversight |
| 84 | Psychedelics as a last resort vs. first-line treatment |
| 85 | Liability and legal risks |
| 86 | Expectations vs. reality |
| 87 | Fear vs. excitement |
| 88 | Agency in the experience |
| 89 | Trust in the therapist |
| 90 | Skepticism vs. belief |
| 91 | Identity shifts |
| 92 | Spiritual vs. scientific framing |
| 93 | Ego dissolution |
| 94 | Need for multiple sessions |
| 95 | Readiness for transformation |
| 96 | Fear of resurfacing trauma |
| 97 | Integration challenges |
| 98 | Self-perception of healing |
| 99 | Long-term impact on mental health |
| 100 | Comparison to other treatments |
| 101 | Support system |
| 102 | Desire for future use |
| 103 | Feelings about altered consciousness |
| 104 | Post-session worldview changes |
| 105 | Concerns about safety |
| 106 | Support role |
| 107 | Fear of personality changes |
| 108 | Trust in medical professionals |
| 109 | Stigma and secrecy |
| **Emotion/Feeling (71 codes)** | |
| 1 | Fear |
| 2 | Anxiety |
| 3 | Guilt |
| 4 | Shame |
| 5 | Hope |
| 6 | Relief |
| 7 | Joy |
| 8 | Awe |
| 9 | Gratitude |
| 10 | Sadness |
| 11 | Grief |
| 12 | Regret |
| 13 | Anger |
| 14 | Frustration |
| 15 | Resentment |
| 16 | Compassion |
| 17 | Love |
| 18 | Connectedness |
| 19 | Trust |
| 20 | Vulnerability |
| 21 | Empowerment |
| 22 | Confusion |
| 23 | Euphoria |
| 24 | Disgust |
| 25 | Loneliness |
| 26 | Despair |
| 27 | Doubt |
| 28 | Endurance |
| 29 | Social Pressure |
| 30 | Sense of Duty |
| 31 | Filial Dilemma |
| 32 | Fear of Trouble |
| 33 | Resignation |
| 34 | Politeness |
| 35 | Bittersweet Pain |
| 36 | Sense of Security |
| 37 | Warmth of Human Connection |
| 38 | Admiration Without Jealousy |
| 39 | Silent Endurance |
| 40 | Small Happiness |
| 41 | Fear of Being Ridiculed |
| 42 | Awkward but Polite |
| 43 | Self-Deprecation |
| 44 | Blessing in Disadvantage |
| 45 | Concern for Nation and People |
| 46 | Cultural Anxiety |
| 47 | Humble Pride |
| 48 | Social Debt |
| 49 | Expected but Misunderstood |
| 50 | Complain but Keep Going |
| 51 | Resilience |
| 52 | Acceptance |
| 53 | Surrender |
| 54 | Professional Detachment |
| 55 | Moral Distress |
| 56 | Burnout |
| 57 | Clinical Confidence |
| 58 | Countertransference |
| 59 | Therapeutic Hope |
| 60 | Compassion Fatigue |
| 61 | Frustration with Systemic Barriers |
| 62 | Helplessness |
| 63 | Disorientation |
| 64 | Catharsis |
| 65 | Breakthrough |
| 66 | Reluctance |
| 67 | Emotional Exhaustion |
| 68 | Guilt for Self-Care |
| 69 | Empathic Bond |
| 70 | Anticipatory Grief |
| 71 | Role Strain |
| **Change of (68 codes)** | |
| 1 | characters |
| 2 | personality |
| 3 | perspective |
| 4 | belief |
| 5 | habit |
| 6 | value |
| 7 | attitude |
| 8 | knowledge |
| 9 | concern |
| 10 | expectation |
| 11 | daily routine |
| 12 | time percpetion |
| 13 | visual perception |
| 14 | speace orientation/perception |
| 15 | bodily feelings |
| 16 | preference |
| 17 | worldview |
| 18 | relationship |
| 19 | mindset |
| 20 | identity |
| 21 | social identity |
| 22 | cultural identity |
| 23 | self-image |
| 24 | understanding |
| 25 | faith |
| 26 | goal |
| 27 | purpose |
| 28 | mission |
| 29 | dharma |
| 30 | philosophy |
| 31 | lifestyle |
| 32 | fashion style |
| 33 | aesthetic sense |
| 34 | working style |
| 35 | career |
| 36 | thinking pattern |
| 37 | gender identity |
| 38 | sexual orientaion |
| 39 | sexual practices |
| 40 | suicidal intention |
| 41 | emotional response |
| 42 | emotional response toward oneself |
| 43 | emotional response toward others |
| 44 | dependency on external validation |
| 45 | ways of interacting with family members |
| 46 | interpretation of memory |
| 47 | stress management |
| 48 | perception of reality |
| 49 | self-reflective thinking |
| 50 | sleep pattern |
| 51 | eating pattern |
| 52 | addictive substance |
| 53 | psychological dependency |
| 54 | slef-control |
| 55 | connection with people |
| 56 | connection with non-human animals |
| 57 | connection with nature |
| 58 | connection with supernatural beings |
| 59 | connection with religious gods |
| 60 | connection with plants |
| 61 | connection with children |
| 62 | connection with family |
| 63 | connection with deities |
| 64 | connection with ancestors |
| 65 | connection with a community |
| 66 | connection with strangers |
| 67 | connection with the earth |
| 68 | connection with the cosmos |
| **Financial/Economic Influence from (18 codes)** | |
| 1 | Price Manipulation |
| 2 | Synthetic Drug Substitution |
| 3 | Government Tax Policies |
| 4 | Funding Priorities |
| 5 | Branding & Commercialization |
| 6 | Medical Capitalization |
| 7 | Standardization of Therapy |
| 8 | Insurance Company Influence |
| 9 | Commercialization of Spiritual Gurus |
| 10 | Short-Term Profit Focus |
| 11 | Overharvesting of Natural Resources |
| 12 | Large-Scale Cultivation |
| 13 | Replacement of Indigenous Traditions |
| 14 | Economic Bubbles |
| 15 | Frequent Consumption |
| 16 | Investment Scams |
| 17 | discrimination at workplace |
| 18 | Risks of Black Market Transactions |
| **Value of (50 codes)** | |
| 1 | religous faith |
| 2 | mysticism |
| 3 | metaphysical commitments |
| 4 | fianaical evaluation |
| 5 | anti-drugs |
| 6 | evidence-based medicine |
| 7 | value-based practice |
| 8 | patient-centered care |
| 9 | resarch performance |
| 10 | patient outcome reports |
| 11 | rapport |
| 12 | Therapeutic alliance |
| 13 | controlled variables |
| 14 | patient autonomy |
| 15 | standardization |
| 16 | Beneficence |
| 17 | Non-maleficence |
| 18 | Justice |
| 19 | Confidentiality |
| 20 | Authenticity |
| 21 | Cultural Competence |
| 22 | Professional Boundaries |
| 23 | Clinical Competence |
| 24 | Empowerment |
| 25 | Non-judgmental Acceptance |
| 26 | Clinical Excellence |
| 27 | Patient Safety |
| 28 | Scientific Integrity |
| 29 | Compassionate Care |
| 30 | Professional Responsibility |
| 31 | Time Management |
| 32 | Diagnostic Accuracy |
| 33 | Cost Consciousness |
| 34 | Team Collaboration |
| 35 | Clear Communication |
| 36 | Being Heard |
| 37 | Timely Access |
| 38 | Respectful Treatment |
| 39 | Quality Care |
| 40 | Cost Transparency |
| 41 | Personal Attention |
| 42 | Pain Management |
| 43 | Regular Updates |
| 44 | Involvement in Care Planning |
| 45 | Support and Resources |
| 46 | Work-Life Balance |
| 47 | Financial Guidance |
| 48 | Emotional Recognition |
| 49 | Clear Instructions |
| 50 | Cultural Respect |
| **Cultural Influence on (120 codes)** | |
| 1 | Religious/Spiritual Background |
| 2 | Current Religious Affiliation |
| 3 | Religious History |
| 4 | Spiritual Practices |
| 5 | Religious Community Involvement |
| 6 | Mystical Experiences |
| 7 | Religious Authority Attitudes |
| 8 | Sacred Text Interpretation |
| 9 | Religious Dietary Practices |
| 10 | Religious Holidays/Ceremonies |
| 11 | Interfaith Experiences |
| 12 | Religious Coping Mechanisms |
| 13 | Spiritual Growth Journey |
| 14 | Religious Social Network |
| 15 | Religious Conflicts |
| 16 | Spiritual Healing Beliefs |
| 17 | Traditional Medicine Practices |
| 18 | Traditional Medicine Experience |
| 19 | Chinese Medicine Practices |
| 20 | Chinese Medicine Experience |
| 21 | Traditional Korean Medicine |
| 22 | Japanese Kampo Medicine |
| 23 | Tibetan Medicine |
| 24 | Mongolian Traditional Medicine |
| 25 | Amazonian Plant Medicine |
| 26 | Andean Traditional Medicine |
| 27 | Native American Medicine |
| 28 | African Indigenous Medicine |
| 29 | Australian Aboriginal Healing |
| 30 | Ayurvedic Medicine |
| 31 | Siddha Medicine |
| 32 | Unani Medicine |
| 33 | Persian Traditional Medicine |
| 34 | Islamic Prophetic Medicine |
| 35 | Greek & Roman Humoral Medicine |
| 36 | Medieval European Herbalism |
| 37 | Nordic Shamanic Healing |
| 38 | Russian Folk Medicine |
| 39 | Polish & Ukrainian Herbal Healing |
| 40 | Curanderismo |
| 41 | Santería & Afro-Caribbean Healing |
| 42 | Healing Modalities Used |
| 43 | Traditional Healer Relationships |
| 44 | Plant Medicine History |
| 45 | Integration of Healing Systems |
| 46 | Family Traditional Medicine |
| 47 | Traditional Diagnostic Methods |
| 48 | Traditional Treatment Preferences |
| 49 | Seasonal Health Practices |
| 50 | Energy Medicine Beliefs |
| 51 | Traditional Medicine Knowledge |
| 52 | Herbal Medicine Use |
| 53 | Body Work Practices |
| 54 | Traditional Health Philosophy |
| 55 | Medicine Preparation Methods |
| 56 | Mental Health Cultural Attitudes |
| 57 | Mental Health Stigma |
| 58 | Help-Seeking Behaviors |
| 59 | Treatment Preferences |
| 60 | Family Mental Health Views |
| 61 | Community Mental Health Resources |
| 62 | Cultural Explanatory Models |
| 63 | Traditional vs Modern Treatment |
| 64 | Mental Health Language |
| 65 | Recovery Expectations |
| 66 | Social Support Role |
| 67 | Mental Health Prevention |
| 68 | Emotional Expression Norms |
| 69 | Legal and Policy Environment |
| 70 | Drug Policy Attitudes |
| 71 | Medicalization vs. Decriminalization |
| 72 | Trust in Governmental Health Regulations |
| 73 | Historical Psychedelic Policies |
| 74 | Institutional Barriers |
| 75 | Religious and Spiritual Contexts |
| 76 | Sacred vs. Secular Healing |
| 77 | Shamanic vs. Scientific Approaches |
| 78 | Reincarnation and Karma Views |
| 79 | Purification Rituals |
| 80 | Interpersonal and Community Norms |
| 81 | Collectivist vs. Individualist Healing |
| 82 | Family Approval of Therapy |
| 83 | Community Stigma and Reputation |
| 84 | Expectations for Emotional Disclosure |
| 85 | Healing as a Social Obligation |
| 86 | Healthcare and Mental Health Norms |
| 87 | Preference for Holistic vs. Biomedical Models |
| 88 | Role of Traditional Psychiatry |
| 89 | Doctor-Patient Power Dynamics |
| 90 | Trust in Therapy as a Concept |
| 91 | Pathologization vs. Expansion of Consciousness |
| 92 | Language and Meaning-Making |
| 93 | Cultural Metaphors for Healing |
| 94 | Linguistic Constraints in Expressing Psychedelic Experiences |
| 95 | Narrative Structures of Psychedelic Journeys |
| 96 | Naming and Framing of Psychedelic Effects |
| 97 | Gender and Psychedelic Therapy |
| 98 | Gendered Expectations in Therapy |
| 99 | Safety Concerns for Women and Marginalized Groups |
| 100 | Role of Male vs. Female Facilitators |
| 101 | Cultural Perceptions of Feminine and Masculine Energies in Healing |
| 102 | Economic and Accessibility Factors |
| 103 | Affordability of Psychedelic Therapy |
| 104 | Elite vs. Popular Access to Psychedelics |
| 105 | Insurance and Financial Support for Therapy |
| 106 | Psychedelics as a Commodity vs. a Right |
| 107 | Perceptions of Risk and Control |
| 108 | Fear of Losing Control |
| 109 | Cultural Boundaries Around Death and Rebirth Experiences |
| 110 | Intergenerational Fear of Drug Use |
| 111 | Addiction Stigma in Psychedelic Use |
| 112 | Altered States and Creativity |
| 113 | Psychedelics and Artistic Traditions |
| 114 | Dreaming and Visionary Experiences |
| 115 | Music and Psychedelic Therapy |
| 116 | Cultural Archetypes Encountered in Psychedelic States |
| 117 | Intergenerational Perspectives on Healing |
| 118 | Youth vs. Elder Views on Psychedelics |
| 119 | Healing Ancestral Trauma |
| 120 | Community Elders as Gatekeepers |
| **Societal/Relational on (47 codes)** | |
| 1 | partner |
| 2 | family |
| 3 | friend |
| 4 | pet |
| 5 | colleague |
| 6 | boss |
| 7 | caregiver |
| 8 | doctor |
| 9 | healthcare provider |
| 10 | pharamacist |
| 11 | sex partner |
| 12 | child |
| 13 | neighbor |
| 14 | customer |
| 15 | subordinate |
| 16 | team member |
| 17 | enemy |
| 18 | rival |
| 19 | teacher |
| 20 | classmate |
| 21 | roommate |
| 22 | stranger |
| 23 | Legal and Regulatory Status |
| 24 | Cultural Attitudes and Stigma |
| 25 | Healthcare Accessibility and Equity |
| 26 | Integration into Mainstream Medicine |
| 27 | Public Health Considerations |
| 28 | Insurance and Cost Coverage |
| 29 | Ethical Considerations |
| 30 | Family and Social Support |
| 31 | Therapeutic Alliance |
| 32 | Community Perceptions |
| 33 | Potential for Interpersonal Conflicts |
| 34 | Integration into Daily Life |
| 35 | Cultural and Religious Attitudes |
| 36 | Social Stigma and Judgment |
| 37 | Medical Legitimacy and Trust |
| 38 | Media Representation and Public Discourse |
| 39 | Workplace and Professional Impact |
| 40 | Social Identity and Belonging |
| 41 | Community and Integration Support |
| 42 | Policy and Ethical Considerations |
| 43 | Emotional Reactions and Support |
| 44 | Trust in Healthcare Providers |
| 45 | Changes in Relationship Dynamics |
| 46 | Caregiving Responsibilities |
| 47 | Expectations for Outcomes |
| **Focus on X (37 codes)** | |
| 1 | Focus on self-awareness |
| 2 | Focus on emotional regulation |
| 3 | Focus on trauma processing |
| 4 | Focus on memory recall |
| 5 | Focus on altered states of consciousness |
| 6 | Focus on existential thoughts |
| 7 | Focus on self-identity reconstruction |
| 8 | Focus on thought looping |
| 9 | Focus on sensory enhancement |
| 10 | Focus on perception of time |
| 11 | Focus onbodily sensations |
| 12 | Focus on dream-like states |
| 13 | Focus on connection with nature |
| 14 | Focus on connection with the universe |
| 15 | Focus on interpersonal relationships |
| 16 | Focus on spiritual experiences |
| 17 | Focus on divine encounters |
| 18 | Focus on motivation shifts |
| 19 | Focus on addiction recovery |
| 20 | Focus on risk-taking behavior |
| 21 | Focus on altered perception |
| 22 | Focus on self-reflection |
| 23 | Focus on spiritual awakening |
| 24 | Focus on ego dissolution |
| 25 | Focus on emotional catharsis |
| 26 | Focus on trauma resurfacing |
| 27 | Focus on suicidal ideation |
| 28 | Focus on anxiety relief |
| 29 | Focus on depressive episodes |
| 30 | Focus on emotional numbness |
| 31 | Focus on emotional waves |
| 32 | Focus on energy flow |
| 33 | Focus on sensory overload |
| 34 | Focus on breaking social norms |
| 35 | Focus on dopamine system alterations |
| 36 | Focus on substance dependence |
| 37 | Focus on enhanced mindfulness |
