# Supplementary material for: Feeling safe: an empirical-ethical analysis of vulnerability in (es)ketamine treatment
Source: BMC Med Ethics. 2026 Oct 8;27:218. doi: 10.1186/s12910-026-01637-7 (PMC13659964; doi:10.1186/s12910-026-01637-7)
Supplement: Supplementary file 8 [file 12910_2026_1637_MOESM7_ESM.docx]

**Additional file 7: Codebook for the category system**

*Feeling Safe: An Empirical-Ethical Analysis of Vulnerability in (Es)ketamine Treatment*

This file reproduces the codebook for the full category system. Part A gives the eleven main categories analysed in the study of safety experience together with the 36 subcategories developed inductively within them; part B gives the nine remaining main categories, which were applied to the corpus but were not selected for this sub-analysis and within which no subcategories were developed. For each category the file gives the definition, whether it was derived deductively from the project code system (main categories, reproduced in Additional file 6) or inductively from the material (subcategories), the rule under which it was applied, and an anchor example with its source. Anchor examples are identified by the anonymized participant code and the paragraph number of the transcript. The codebook was formulated and applied in German, apart from the interviews conducted in English, and has been translated in full for this file; category designations follow the terminology used in the manuscript wherever a category is named there. Anchor examples from German-language interviews are given in translation; those from interviews conducted in English are reproduced verbatim and marked [English]. Coding frequencies for these categories are reported in Additional file 8.

| **Category** | **Definition** | **Origin** | **Coding rule: the category is coded when…** | **Anchor example (quotation with source)** |
| --- | --- | --- | --- | --- |
| **A. The eleven main categories analysed in the study of safety experience, with the 36 subcategories developed inductively within them** | | | | |
| **1. Roles of or Relationship with Healthcare and Medical Professionals** | Records interactions with clinicians in the context of the therapy, and thoughts about the therapeutic relationship. | Main category: deductive | …descriptions of interpersonal conduct, of presence, or of the role of the treating staff occur. | “The psychiatrist sat right next to me… He is so nice, but I hardly knew him. … I did not actually speak a word with him during the session.” (IVI_05:73) |
| 1.1 Therapeutic presence vs. administrative distance | Records the quality of attention (resonance vs. distraction through bureaucracy). | Subcategory: inductive | …it is at issue whether clinicians are available for resonance or are distracted by work on a laptop. | “… he was sitting there at the laptop the whole time … I think he was also checking emails or something.” (IVI_01:67) |
| 1.2 Active physical grounding and crisis management | Targeted actions in moments of deep dissociation or anxiety. | Subcategory: inductive | …active interventions such as hand-holding or reassuring words are mentioned. | “… the first time I asked him to hold my hand because I was like, do I exist still?” (IVI_02:31) [English] |
| 1.3 Professional authority as a basis of trust | Stabilizing effect of medical competence and credibility. | Subcategory: inductive | …trust is based explicitly on medical expertise or on clinical safeguarding. | “… that feeling of medical authority gave me kind of like a reassurance basically.” (S_05:23) [English] |
| 1.4 Quality of psychoeducational preparation and integration (“landing aid”) | Extent of active preparation and of accompaniment back into everyday life (integration). | Subcategory: inductive | …the transition out of the setting, or the absence of a closing conversation, is evaluated. | “… you are basically adjusted and discharged by the clock.” (IVI_04:45) |
| 1.5 Economization and depersonalization | Treatment of patients primarily as a “service product” or a “case number”. | Subcategory: inductive | …a “conveyor-belt feeling” or time pressure in the handling of the treatment is described. | “I did have the feeling that I am rather part of a machinery … I am a product …” (IVI_01:124) |
| 1.6 Credibility and expertise on equal footing | The need of informed patients to be recognized as equal partners. | Subcategory: inductive | …patients question or validate professional competence in dialogue. | “Well, you have really looked into it, you are a proper expert [sarcastic].” (IVI_01:116) |
| 1.7 Wish for clinicians’ own experience and empathy | The conviction that clinicians should ideally have had the experience themselves. | Subcategory: inductive | …the necessity of an authentic point of reference on the part of the treating staff is emphasized. | “… that someone who has also been through it themselves … because they simply understand things better …” (IVI_01:25) |
| 1.8 Interpersonal warmth vs. functional routine | The polarity between emotional warmth and purely procedural handling. | Subcategory: inductive | …the emotional quality (sympathy vs. technical measurement) is evaluated. | “I hold out my arm, he measures the blood pressure, does this and that, and it is done. Otherwise I have no interaction.” (S_06:71) |
| 1.9 Anticipatory trust through institutional or personal continuity | Security arising from long-standing prior treatment or familiarity with the institution. | Subcategory: inductive | …security rests on a stable basis established before the session. | “Exactly, I had known them all for quite some time and because of that I actually had rather good trust …” (S_03:19) |
| **2. Influences of Treatment Environment (Set and Setting)** | Explains how the spatial frame supports or endangers the sense of safety. | Main category: deductive | …set and setting are described concretely in relation to the experience of therapy. | “I feel comfortable, that is quite clear … of course I also know the room, I am lying down, I am wrapped up in the blanket …” (IVI_03:33) |
| 2.1 Acoustic framing (disruptive noise vs. music and shielding) | The role of ambient noise as against the use of music as shielding. | Subcategory: inductive | …noise (doors slamming) or the effect of the playlist is at issue. | “… if doors are slamming now and someone is shouting around, then for me that is simply an immediate trigger of anxiety.” (IVI_04:105) |
| 2.2 Biographical triggers and retraumatization through the clinical setting | Reactivation of earlier trauma through elements of the hospital environment. | Subcategory: inductive | …elements such as treatment couches or smells are experienced as threatening. | “The couch really was such … I do not want to say that this is how Montesquieu felt on the scaffold …” (IVI_01:77) |
| 2.3 Physiological security (comfort and re-entry rituals) | Material and sensory aspects that promote relaxation (beds, blankets, tea). | Subcategory: inductive | …physical wellbeing arising from the furnishings is described. | “… we drank tea every time after the therapy … that also gave one such a feeling of wellbeing.” (IVI_05:81) |
| 2.4 Spatial symbols of safety and availability | Minimal spatial gestures of availability. | Subcategory: inductive | …details such as a (half) open door are named as a factor of safety. | “And they leave the door like slightly open. That also kind of gives a sense of safety …” (S_05:51) [English] |
| 2.5 Social setting (privacy vs. interference from fellow patients) | Effects of other people (fellow patients) on safety. | Subcategory: inductive | …social density or disturbance by third parties is at issue. | “Then I am standing there with a hundred people in the corridor … but you actually want to stay in your bubble.” (IVI_04:51) |
| 2.6 Visual design and lighting | Visual atmosphere (brightness, choice of colour, eye masks, and so on). | Subcategory: inductive | …the visual environment influences the sense of safety or the intensity of the experience. | “The ceiling … has … tiny holes. And … visually, it’s more engaging … outside there’s the sun blocker curtains … So it’s a dim atmosphere that helps … having a calm environment.” (S_05:55) [English] |
| **3. Evaluation of Psychedelic Treatment** | Retrospective evaluation of the experience with the substance, the setting or the set. | Main category: deductive | …summarizing statements about success, failure or safety occur. | “… the difference compared with before the treatment is enormous, yes.” (S_02:65) |
| 3.1 Retrospective experience of safety | Evaluation of how safe or how threatened the person felt during the process. | Subcategory: inductive | …trust in the monitoring, or in being caught if something went wrong, is summed up. | “That there is someone to turn to … that in any case, should anything happen, … someone is there who could intervene if necessary or could catch me …” (S_01:21) |
| 3.2 Subjective treatment success vs. destabilization | The contrast between symptom relief and negative long-term consequences. | Subcategory: inductive | …the durability of the effect, or a psychological instability, is at issue. | “… now it is almost five months later … it has become … rather such that it has a retraumatizing effect …” (IVI_01:108) |
| **4. Psychological Outlook toward Psychedelic Treatment** | Inner stance and expectations before the start of treatment (set). | Main category: deductive | …initial skepticism, hope or confidence is described. | “And that was then a bit like the straw to clutch at, yes.” (S_02:16) |
| 4.1 Initial trust in the medical system | Confidence in the professional frame before contact. | Subcategory: inductive | …the stabilizing effect of medical authority is named. | “… it gave me a bit more confidence in that, like, oh, this is like a professional medical situation.” (S_05:23) [English] |
| 4.2 Skepticism and aversion | A critical stance, or one shaped by negative prior experience. | Subcategory: inductive | …a defensive stance toward the method or the place is articulated. | “… my whole basic attitude toward the entire place was actually, I do not want to come back here …” (IVI_01:77) |
| 4.3 Hope invested in a “last straw” | A decision taken out of severe treatment resistance and desperation. | Subcategory: inductive | …the treatment is described as the last remaining option. | “… I am doing this now because there is nothing else left.” (S_02:21) |
| **5. Personal and Psychological Concerns about Psychedelic Treatment** | Concerns about psychological change or loss of control, and about physical risks. | Main category: deductive | …fears of “madness”, of ego death, or of physical risk are named. | “… if my demons would just come out and eat me.” (IVI_02:23) [English] |
| 5.1 Psychological and existential fears of crisis | Concerns about the loss of cognitive control, or about annihilation. | Subcategory: inductive | …fear of dying, of psychosis, or of irreversible dissolution of the self is named. | “The bad experiences, the feelings I had there, were pure fear. Fear of death, I would say …” (IVI_05:61) |
| 5.2 Physical concerns | Concerns about somatic reactions or procedures. | Subcategory: inductive | …fears of side effects (blood pressure) or fear of needles are articulated. | “And that is completely impossible for me, I am afraid of needles.” (IVI_04:33) |
| **6. Psychedelic Experiences** | Phenomenology of the acute effect of the substance during the session. | Main category: deductive | …phenomena such as loss of time or altered perception are described. | “… that I could, as it were, watch my brain taking itself apart a little.” (IVI_01:53) |
| 6.1 Deep dissociation (K-hole / ego death) | States of total dissolution of the self and loss of time and space. | Subcategory: inductive | …the loss of any reference to reality or to the body is described. | “And then I felt like I was dying, what they call the ego death … I lost the concept of self, of reality.” (IVI_02:29) [English] |
| 6.2 Distressing experiences (bad trip) | Acute reactions of anxiety and panic during the effect. | Subcategory: inductive | …feelings of “hell” or painful biographical content are named. | “… it felt like walking through hell.” (IVI_05:61) |
| 6.3 Mild relaxation | Experiences of physical lightness or wellbeing. | Subcategory: inductive | …pleasant perceptions without threat are described. | “… I find this treatment … incredibly mild … quite relaxed and good.” (IVI_03:19) |
| **7. Patient Journey** | Narrative account of the illness biography and of treatment pathways. | Main category: deductive | …biographical trauma or negative experiences within the health care system are reported. | “… over the last years I have had a great deal of bad experience in hospital.” (S_04:41) |
| 7.1 Biographical traumatization | Distressing experiences (childhood, violence) outside the clinical system. | Subcategory: inductive | …accounts of attachment disturbance serve as the basis of vulnerability. | “Because my traumatizations went so deep, from childhood …” (IVI_01:7) |
| 7.2 Institutional retraumatization | Negative experiences within the health care system. | Subcategory: inductive | …earlier experiences in clinics cause the present setting to act as a trigger. | “… I really had to prepare myself inwardly for having to be in those rooms twice a week to begin with.” (S_04:39) |
| **8. Roles of or Relationship with Non-Medical Individuals** | Social resources during treatment (family, friends). | Main category: deductive | …private companions as a pillar of safety, or the attitude of the social environment, are at issue. | “… I then met my mate … that helped a bit.” (IVI_01:67) |
| 8.1 Private companions as a pillar of safety | Active involvement of trusted persons in the process. | Subcategory: inductive | …friends or partners are named as a resource for stability or logistics. | “… that they are there in the treatment room … That is another such additional pillar of safety.” (S_01:74) |
| 8.2 Attitudes in the immediate social environment (family and friends) | The concrete stance of significant others toward the treatment. | Subcategory: inductive | …the influence of personal exchange on the experience is described. | “Well, my mother … my siblings were a bit shocked … But I then informed them … They were quite enthusiastic.” (IVI_05:51) |
| **9. Knowledge of Psychedelic Treatment** | Knowledge and models concerning how the therapy works. | Main category: inductive | …patients express specialist knowledge, knowledge drawn from media, or their own experience. | “… they can make your brain a little more plastic … and help you rewire some parts of it.” (IVI_02:5) [English] |
| 9.1 Theoretical specialist knowledge (“expert patient”) | Deep understanding based on literature or profession (for example neuroplasticity). | Subcategory: inductive | …terms such as default mode network are used correctly. | “My understanding was … that psychedelics … increase the neuroplasticity of the brain … to turn down the default mode network … to calm the amygdala …” (IVI_01:13) |
| 9.2 Information drawn from media | Knowledge from documentaries, podcasts or the internet. | Subcategory: inductive | …information is explicitly traced back to documentaries or podcasts. | “I heard on a podcast about, in the US, somebody having a friend who’s doing this …” (S_05:17) [English] |
| 9.3 Experiential knowledge (psychonauts) | Understanding derived from earlier (legal or illegal) personal use. | Subcategory: inductive | …effects are compared with earlier personal experiences of intoxication. | “Well, I … knew it, because I had taken LSD before and MDMA quite often back then.” (IVI_05:7) |
| **10. Influences of Treatment Costs** | Mention of cost as a factor in the decision or in the experience. | Main category: inductive | …economic pressure, or a sense of entitlement arising from it, is named. | “And that does influence me, because … a session there would have cost 3,000 euros. That would have put me under pressure …” (S_01:86) |
| 10.1 Sense of entitlement arising from self-payment | Heightened expectation of clinicians on account of private funding. | Subcategory: inductive | …the level of the price is linked to the expected quality of care. | “… that is also the expectation, if I am paying that much money … That contributed to my doubting everything.” (IVI_01:116) |
| 10.2 Economic pressure | Burden and stress arising from funding (for example by relatives). | Subcategory: inductive | …feelings of guilt toward those funding the treatment, or pressure of cost, are named. | “One did feel under pressure, one has to transfer the money after all … And that is not a small amount.” (IVI_05:109) |
| **11. Suggestions for Psychedelic Treatment** | Concrete wishes for improvement of the procedure or the setting. | Main category: inductive | …wishes for better integration or for structural change are expressed. | “I think that would simply be a gentler landing. And also that one keeps the feeling, or is not immediately spat out again like that.” (IVI_04:47) |
| 11.1 Improving the “landing aid” | Wishes for improved therapeutic accompaniment on “arriving back”. | Subcategory: inductive | …a need for closing conversations or for emotional holding is articulated. | “… that one does not even take five or ten minutes to talk about what has just happened …” (IVI_04:45) |
| 11.2 Structural suggestions for improvement | Suggestions concerning physical arrangements or clinical procedures. | Subcategory: inductive | …suggestions for furniture (a bed), lighting or quiet are made. | “… a perfect setting would be a room with a bed as well. Because I find the couch very hard.” (IVI_04:47) |
| **B. The nine remaining main categories of the system, applied to the corpus but not selected for this sub-analysis** | | | | |
| **12. Influences of Cultural, Social and Political Environment** | Sociocultural influences on the decision for or against treatment, such as social media, news, podcasts or blogs, as distinct from the influence of individual social contacts and significant others; also includes remarks on features of the health system of the country. | Main category: deductive | …influences of the wider social, cultural or political environment on the decision or on the experience are described. | “That is unfortunately how it is in our system, which I find a real pity. That is why I always think it is good to speak in person, because often it now goes by email or whatever.” (IVI_04:129) |
| **13. Intrapersonal Changes after Psychedelic Treatment** | Lasting changes in experience, including side effects persisting beyond the acute effects, and psychological changes in attitudes or behaviour. Not for purely physiological changes such as changes in heart rate. | Main category: deductive | …a lasting change in experience, attitude or behaviour following treatment is reported. | “Yes, absolutely. I am perhaps not yet at the level where I would like to be, but the difference compared with before the treatment is enormous, yes.” (S_02:65) |
| **14. Goals of Psychedelic Treatment** | What the patient wants, or wanted, to achieve through the treatment. | Main category: deductive | …aims or hoped-for outcomes of the treatment are named. | “A little — this may be a fallacy — but a little away from the chemistry, that is, away from the tablets.” (S_01:31) |
| **15. Background Knowledge** | Background knowledge or beliefs reported by the patient that are not related to psychedelics, including medical expert knowledge and knowledge of psychopathology and neuroscience. | Main category: deductive | …knowledge or belief is reported that bears on the illness or its treatment but not specifically on psychedelics. | “Actually I am always the one who knows most about it, both about my illness history and about the treatment methods, and nobody could ever tell me much on that.” (IVI_01:41) |
| **16. Social, Ethical and Political Concerns of Psychedelic Treatment** | Concerns regarding legal consequences, social reactions and ethical failure related to the decision to undergo psychedelic or ketamine treatment. | Main category: deductive | …legal, social or ethical concerns about undergoing the treatment are voiced. | “I do not have a circle of friends and have never had much to do with psychedelic drugs myself in the past, so I did not know it from my environment at all, nor in my family.” (S_02:43) |
| **17. Interpersonal Changes after Psychedelic Treatment** | Explicit mention that a relationship has changed as a consequence of treatment, as distinct from intrapersonal change that leads to behavioural change on the patient's side. | Main category: deductive | …a change in a specific relationship is attributed to the treatment. | “There is not much family in my case. I have only a brother left. And of course he was astonished, because his wife is a doctor as well. … But then I explained that it is esketamine and so on.” (IVI_04:69) |
| **18. Social, Ethical or Political Changes after Psychedelic Treatment** | Changes in one's perspective on social, ethical or political issues. | Main category: deductive | …a shift in social, ethical or political outlook following treatment is described. | “I am surprised that society is opening up more and more. As I said, legalization of drugs. … I also know many people who are much more relaxed when they smoke one.” (IVI_04:79) |
| **19. Spiritual, Religious, Philosophical and Metaphysical Changes after Psychedelic Treatment** | Change of perspective after treatment on religious questions, on the nature of the world and of reality, on what there is and on the meaning of what there is. | Main category: deductive | …a shift in spiritual, religious, philosophical or metaphysical outlook following treatment is described. | “During these dissociation trips, or whatever one wants to call them, there is always a bit of this theme of transience, so a lot of it is about death. Not always entirely in a negative sense …” (S_02:69) |
| **20. Spiritual, Religious, Philosophical and Metaphysical Concerns of Psychedelic Treatment** | Concerns of a spiritual, religious, philosophical or metaphysical kind regarding the treatment. | Main category: deductive | …concerns of this kind about undergoing the treatment are voiced. | “So, ethical, no, because I know it’s sourced legally, so I didn’t feel in any way about this, and I don’t think using hallucinogenics or any type of drug is inherently bad.” (IVI_02:26) [English] |
