# Supplementary material for: Feeling safe: an empirical-ethical analysis of vulnerability in (es)ketamine treatment
Source: BMC Med Ethics. 2026 Oct 8;27:218. doi: 10.1186/s12910-026-01637-7 (PMC13659964; doi:10.1186/s12910-026-01637-7)
Supplement: Supplementary file 9 [file 12910_2026_1637_MOESM8_ESM.docx]

**Additional file 9: Case-to-type relationships for the mechanism-of-action typology**

*Feeling Safe: An Empirical-Ethical Analysis of Vulnerability in (Es)ketamine Treatment*

This file reports, in de-identified form, the value of each case on the five dimensions of the feature space and its assignment to a type for the typology of mechanism-of-action models described in the manuscript, together with a note on the modes of experiencing safety. Participant designations are the anonymized codes used throughout the manuscript: E denotes an interviewed clinician, IVI a patient treated by intravenous infusion and S a patient treated with intranasal esketamine (Spravato®). Case numbers are those used in the manuscript and in Additional file 8. The dimensions across which cases were characterized in the type-building analysis are documented in Additional file 10.

The mechanism-of-action types are reported as provisional interpretive patterns rather than as validated classifications. Types were constructed polythetically, so that cases are grouped by overall similarity across the dimensions rather than by any single shared criterion; no case matches its type on every dimension, and the types are internally heterogeneous. Cases combining features of more than one type were assigned on the balance of dimensions and, where this remained unclear, on the account of the mechanism of action itself. Cases that could not be characterized across the dimensions would have been left unassigned rather than allocated to the nearest type; in the event all 24 cases could be characterized and none was left unassigned.

**Table A. Models of mechanism of action: case-by-dimension matrix and type assignment**

| **Case** | **Group** | **Mechanistic Understanding** | **Clinical Role and Value** | **Experiential Significance** | **Efficacy Assessment** | **Patient Agency** |
| --- | --- | --- | --- | --- | --- | --- |
| **Type 1 – Catalyst of transformative processes (n = 8)** | | | | | | |
| E_03 | Clinician | Neuroplastic Model | Ambivalent Positioning | Transformative or Insightful | Process Focus | Active Engagement |
| E_04 | Clinician | Neuroplastic Model | Transformative Opportunity / Ambivalent Positioning | Transformative or Insightful | Process Focus | Active Engagement |
| E_06 | Clinician | Neuroplastic Model / Vague or Hybrid Explanations | Ambivalent Positioning | Transformative or Insightful | Process Focus | – |
| E_07 | Clinician | Neuroplastic Model | Ambivalent Positioning | Transformative or Insightful | Acute Relief | Active Engagement |
| E_08 | Clinician | Neuroplastic Model | Ambivalent Positioning | Moderately Meaningful or Ambivalent | Process Focus / Acute Relief | – |
| E_10 | Clinician | Vague or Hybrid Explanations | Ambivalent Positioning | Transformative or Insightful | Process Focus | Active Engagement / Partial Engagement |
| IVI_01 | Patient – infusion | Neuroplastic Model | – | Transformative or Insightful | Process Focus / Acute Relief | – |
| S_09 | Patient – esketamine | Vague or Hybrid Explanations | Transformative Opportunity | Moderately Meaningful or Ambivalent | Process Focus | Partial Engagement |
| **Type 2 – Moments of clarity and relief (n = 8)** | | | | | | |
| E_05 | Clinician | Vague or Hybrid Explanations | Transformative Opportunity / Ambivalent Positioning | Moderately Meaningful or Ambivalent | Symptom Focus | – |
| E_09 | Clinician | Neuroplastic Model / Vague or Hybrid Explanations | Ambivalent Positioning | Transformative or Insightful / Moderately Meaningful or Ambivalent | Process Focus / Acute Relief | – |
| IVI_02 | Patient – infusion | Neuroplastic Model | – | Moderately Meaningful or Ambivalent | Acute Relief | Partial Engagement |
| IVI_03 | Patient – infusion | – | Last-Resort Option | Moderately Meaningful or Ambivalent | Acute Relief | Partial Engagement |
| IVI_04 | Patient – infusion | Vague or Hybrid Explanations / Mechanism Irrelevant or Unknown | Ambivalent Positioning / Last-Resort Option | Moderately Meaningful or Ambivalent | Acute Relief / Symptom Focus | – |
| IVI_05 | Patient – infusion | Vague or Hybrid Explanations / Mechanism Irrelevant or Unknown | Transformative Opportunity / Ambivalent Positioning | Moderately Meaningful or Ambivalent | Symptom Focus | Partial Engagement |
| S_03 | Patient – esketamine | Neuroplastic Model / Vague or Hybrid Explanations | Ambivalent Positioning | Experiential Side Effect | Acute Relief / Symptom Focus | – |
| S_08 | Patient – esketamine | Mechanism Irrelevant or Unknown | Ambivalent Positioning / Last-Resort Option | Moderately Meaningful or Ambivalent | Acute Relief / Symptom Focus | Partial Engagement |
| **Type 3 – Pharmacological symptom management (n = 8)** | | | | | | |
| E_01 | Clinician | Vague or Hybrid Explanations / Mechanism Irrelevant or Unknown | Last-Resort Option | Experiential Side Effect | Symptom Focus | – |
| E_02 | Clinician | – | Last-Resort Option | Experiential Side Effect | Acute Relief / Symptom Focus | – |
| S_01 | Patient – esketamine | – | Ambivalent Positioning / Last-Resort Option | Experiential Side Effect | Symptom Focus | Passive Reception |
| S_02 | Patient – esketamine | Vague or Hybrid Explanations / Mechanism Irrelevant or Unknown | Ambivalent Positioning / Last-Resort Option | Moderately Meaningful or Ambivalent / Experiential Side Effect | Symptom Focus | Partial Engagement |
| S_04 | Patient – esketamine | Mechanism Irrelevant or Unknown | Ambivalent Positioning / Last-Resort Option | Experiential Side Effect | Symptom Focus | – |
| S_05 | Patient – esketamine | – | Ambivalent Positioning / Last-Resort Option | Moderately Meaningful or Ambivalent | – | – |
| S_06 | Patient – esketamine | Vague or Hybrid Explanations | Last-Resort Option | Experiential Side Effect | Symptom Focus | – |
| S_07 | Patient – esketamine | Vague or Hybrid Explanations / Mechanism Irrelevant or Unknown | Ambivalent Positioning / Last-Resort Option | Moderately Meaningful or Ambivalent | – | – |

Cells give the value coded for each case on each dimension of the feature space (Additional file 10, Table A); '–' indicates that the dimension was not addressed in the interview; two values indicate that both were coded. Types were constructed polythetically, so that no case matches its type on every dimension.

**Table B. Distribution of cases across types by participant group and treatment modality**

| **Type** | **Clinicians** | **Patients: infusion** | **Patients: esketamine** | **Total** |
| --- | --- | --- | --- | --- |
| Type 1 – Catalyst of transformative processes | 6 | 1 | 1 | 8 |
| Type 2 – Moments of clarity and relief | 2 | 4 | 2 | 8 |
| Type 3 – Pharmacological symptom management | 2 | 0 | 6 | 8 |
| **Total corpus** | **10** | **5** | **9** | **24** |

Two features of this distribution are noted in section 3.3 of the manuscript. Clinicians are concentrated in Type 1. And no patient treated by infusion was classified as Type 3. Both are reported descriptively; neither is interpreted as an effect of the treatment modality, since in this sample infusion treatment is confounded with the provision of structured integration work and with self-payment, and since with five infusion patients the absence of a type in that subgroup is uninformative.

**C. Modes of experiencing safety**

The three modes of experiencing safety reported in section 3.2.1 of the manuscript (informed skeptic, relationship-oriented truster, autonomous self-regulator) are overlapping interpretive patterns derived from case summaries, not mutually exclusive classifications of participants, and were not produced by formal type construction against a predefined feature space. Most participants combined features of more than one mode, and the modes name recurring patterns rather than groups to which individuals belong. For this reason no case-to-mode assignment is reported; the illustrative cases named in section 3.2.1 of the manuscript indicate which accounts most clearly exemplify each pattern.
