# Supplementary material for: Feeling safe: an empirical-ethical analysis of vulnerability in (es)ketamine treatment
Source: BMC Med Ethics. 2026 Oct 8;27:218. doi: 10.1186/s12910-026-01637-7 (PMC13659964; doi:10.1186/s12910-026-01637-7)
Supplement: Supplementary file 10 [file 12910_2026_1637_MOESM9_ESM.docx]

**Additional file 8: Distribution of codings across the category system**

*Feeling Safe: An Empirical-Ethical Analysis of Vulnerability in (Es)ketamine Treatment*

This file reports the distribution of codings across all 14 patient interviews. Both the main categories and the inductively developed subcategories were applied to the complete patient corpus; no cell is left unretrieved. Part A gives the eleven main categories selected for the analysis of safety experience together with the 36 subcategories developed inductively within them; part B gives the remaining nine main categories of the system, which were applied to the corpus but were not selected for this sub-analysis and within which no subcategories were developed. Counts are given per case and in total. They are reported as context for the qualitative analysis and do not constitute a quantitative weighting in a statistical sense.

Category and subcategory designations are given in English. The subcategories were formulated in German during coding and have been translated here; the wording follows the terminology used in the manuscript wherever a category is named there, and the German originals are given in the codebook (Additional file 7).

| **No.** | **Category / subcategory** | **IVI_01** | **IVI_02** | **IVI_03** | **IVI_04** | **IVI_05** | **S_01** | **S_02** | **S_03** | **S_04** | **S_05** | **S_06** | **S_07** | **S_08** | **S_09** | **Total** |
| --- | --- | --- | --- | --- | --- | --- | --- | --- | --- | --- | --- | --- | --- | --- | --- | --- |
| **A. The eleven main categories selected for the analysis of safety experience, with their inductive subcategories** | | | | | | | | | | | | | | | | |
| **1** | **Roles of or Relationship with Healthcare and Medical Professionals** | **17** | **4** | **7** | **7** | **9** | **10** | **10** | **9** | **5** | **5** | **4** | **5** | **11** | **19** | **122** |
| 1.1 | Therapeutic presence vs. administrative distance (laptop use) | 7 | 0 | 2 | 0 | 1 | 2 | 1 | 4 | 0 | 0 | 0 | 0 | 0 | 1 | 18 |
| 1.2 | Active physical grounding and crisis management | 5 | 1 | 0 | 0 | 1 | 1 | 2 | 0 | 1 | 0 | 0 | 0 | 0 | 0 | 11 |
| 1.3 | Professional authority as a basis of trust | 4 | 0 | 1 | 0 | 1 | 1 | 0 | 0 | 0 | 2 | 0 | 1 | 0 | 0 | 10 |
| 1.4 | Quality of psychoeducational preparation and integration (“landing aid”) | 12 | 1 | 0 | 2 | 1 | 2 | 0 | 2 | 0 | 0 | 1 | 0 | 1 | 4 | 25 |
| 1.5 | Economization and depersonalization | 9 | 0 | 0 | 0 | 0 | 0 | 0 | 0 | 0 | 0 | 0 | 0 | 0 | 1 | 10 |
| 1.6 | Credibility and expertise on equal footing | 5 | 0 | 1 | 0 | 1 | 0 | 0 | 0 | 0 | 0 | 0 | 0 | 1 | 0 | 8 |
| 1.7 | Wish for clinicians’ own experience and empathy | 2 | 0 | 0 | 0 | 0 | 1 | 0 | 0 | 0 | 0 | 0 | 0 | 0 | 0 | 3 |
| 1.8 | Interpersonal warmth vs. functional routine | 4 | 0 | 1 | 0 | 5 | 2 | 0 | 1 | 2 | 1 | 1 | 0 | 2 | 2 | 21 |
| 1.9 | Anticipatory trust through institutional or personal continuity | 1 | 0 | 0 | 1 | 0 | 1 | 0 | 2 | 2 | 1 | 0 | 0 | 0 | 1 | 9 |
| **2** | **Influences of Treatment Environment (Set and Setting)** | **15** | **1** | **2** | **2** | **10** | **11** | **4** | **8** | **4** | **3** | **1** | **2** | **4** | **18** | **85** |
| 2.1 | Acoustic framing (disruptive noise vs. music and shielding) | 2 | 0 | 0 | 2 | 1 | 1 | 1 | 0 | 0 | 1 | 0 | 0 | 0 | 6 | 14 |
| 2.2 | Biographical triggers and retraumatization through the clinical setting | 5 | 0 | 0 | 0 | 0 | 0 | 0 | 0 | 3 | 0 | 0 | 0 | 0 | 1 | 9 |
| 2.3 | Physiological security (comfort and re-entry rituals) | 0 | 1 | 1 | 0 | 0 | 2 | 0 | 1 | 0 | 0 | 2 | 0 | 0 | 2 | 9 |
| 2.4 | Spatial symbols of safety and availability | 2 | 0 | 1 | 0 | 0 | 1 | 0 | 1 | 0 | 2 | 0 | 0 | 0 | 1 | 8 |
| 2.5 | Social setting (privacy vs. interference from fellow patients) | 0 | 0 | 1 | 1 | 0 | 1 | 2 | 0 | 0 | 3 | 2 | 0 | 2 | 1 | 12 |
| 2.6 | Visual design and lighting | 2 | 1 | 0 | 1 | 3 | 0 | 0 | 0 | 0 | 1 | 0 | 0 | 0 | 1 | 9 |
| **3** | **Evaluation of Psychedelic Treatment** | **18** | **1** | **4** | **6** | **15** | **15** | **8** | **9** | **4** | **0** | **4** | **0** | **7** | **23** | **114** |
| 3.1 | Retrospective experience of safety | 1 | 1 | 1 | 0 | 5 | 2 | 0 | 2 | 0 | 1 | 0 | 0 | 1 | 0 | 14 |
| 3.2 | Subjective treatment success vs. destabilization | 2 | 0 | 1 | 2 | 1 | 0 | 1 | 0 | 1 | 0 | 2 | 0 | 2 | 2 | 14 |
| **4** | **Attitude towards Psychedelic Treatment** | **14** | **2** | **7** | **7** | **6** | **12** | **12** | **4** | **4** | **1** | **1** | **4** | **8** | **25** | **107** |
| 4.1 | Initial trust in the medical system | 1 | 0 | 0 | 1 | 1 | 1 | 0 | 0 | 0 | 1 | 0 | 2 | 2 | 1 | 10 |
| 4.2 | Skepticism and aversion | 2 | 1 | 1 | 1 | 0 | 0 | 0 | 0 | 2 | 0 | 2 | 2 | 0 | 1 | 12 |
| 4.3 | Hope invested in a “last straw” | 2 | 0 | 0 | 4 | 0 | 1 | 2 | 1 | 1 | 0 | 0 | 7 | 6 | 0 | 24 |
| **5** | **Personal and Psychological Concerns of Psychedelic Treatment** | **15** | **3** | **1** | **4** | **5** | **10** | **6** | **3** | **3** | **5** | **3** | **1** | **5** | **8** | **72** |
| 5.1 | Psychological and existential fears of crisis | 4 | 3 | 0 | 1 | 3 | 1 | 0 | 0 | 0 | 1 | 3 | 1 | 1 | 0 | 18 |
| 5.2 | Physical concerns | 1 | 0 | 1 | 1 | 0 | 0 | 1 | 0 | 0 | 3 | 3 | 0 | 0 | 2 | 12 |
| **6** | **Psychedelic Experiences** | **5** | **1** | **3** | **7** | **13** | **9** | **2** | **3** | **3** | **1** | **9** | **0** | **6** | **6** | **68** |
| 6.1 | Deep dissociation (K-hole / ego death) | 3 | 1 | 0 | 0 | 1 | 1 | 0 | 0 | 0 | 0 | 1 | 0 | 0 | 0 | 7 |
| 6.2 | Distressing experiences (bad trip) | 1 | 0 | 0 | 0 | 1 | 2 | 1 | 0 | 1 | 0 | 0 | 0 | 0 | 2 | 8 |
| 6.3 | Mild relaxation | 0 | 0 | 1 | 1 | 0 | 1 | 0 | 1 | 0 | 1 | 0 | 0 | 2 | 1 | 8 |
| **7** | **Patient Journey** | **11** | **5** | **7** | **11** | **6** | **5** | **7** | **6** | **8** | **2** | **10** | **10** | **8** | **18** | **114** |
| 7.1 | Biographical traumatization | 3 | 0 | 1 | 0 | 1 | 0 | 0 | 1 | 0 | 0 | 0 | 0 | 0 | 1 | 7 |
| 7.2 | Institutional retraumatization | 3 | 0 | 1 | 0 | 1 | 0 | 0 | 0 | 3 | 0 | 0 | 0 | 0 | 3 | 11 |
| **8** | **Roles of or Relationship with Non-Medical Individuals** | **6** | **2** | **2** | **1** | **3** | **10** | **2** | **1** | **1** | **2** | **3** | **1** | **4** | **2** | **40** |
| 8.1 | Private companions as a pillar of safety | 3 | 0 | 1 | 0 | 0 | 6 | 0 | 0 | 0 | 0 | 0 | 0 | 1 | 0 | 11 |
| 8.2 | Attitudes in the immediate social environment (family and friends) | 1 | 1 | 3 | 1 | 1 | 2 | 1 | 1 | 1 | 1 | 1 | 2 | 1 | 1 | 18 |
| **9** | **Knowledge of Psychedelic Treatment** | **13** | **2** | **3** | **2** | **10** | **9** | **10** | **11** | **4** | **2** | **12** | **7** | **4** | **12** | **101** |
| 9.1 | Theoretical specialist knowledge (“expert patient”) | 1 | 1 | 1 | 0 | 0 | 1 | 0 | 2 | 0 | 0 | 3 | 0 | 1 | 1 | 11 |
| 9.2 | Information drawn from media | 0 | 0 | 0 | 1 | 1 | 1 | 1 | 0 | 1 | 1 | 0 | 5 | 3 | 1 | 15 |
| 9.3 | Experiential knowledge (psychonauts) | 1 | 1 | 0 | 1 | 1 | 1 | 0 | 0 | 0 | 0 | 0 | 0 | 1 | 1 | 7 |
| **10** | **Influences of Treatment Costs** | **12** | **1** | **1** | **1** | **3** | **3** | **1** | **2** | **0** | **0** | **0** | **0** | **3** | **3** | **30** |
| 10.1 | Sense of entitlement arising from self-payment | 2 | 0 | 0 | 0 | 1 | 1 | 0 | 0 | 0 | 0 | 0 | 0 | 0 | 0 | 4 |
| 10.2 | Economic pressure | 2 | 0 | 1 | 0 | 2 | 0 | 0 | 0 | 0 | 0 | 0 | 0 | 0 | 1 | 6 |
| **11** | **Suggestions for Psychedelic Treatment** | **4** | **0** | **0** | **0** | **0** | **0** | **0** | **0** | **1** | **0** | **0** | **0** | **4** | **9** | **18** |
| 11.1 | Improving the “landing aid” | 4 | 0 | 1 | 1 | 1 | 0 | 0 | 0 | 0 | 0 | 0 | 0 | 0 | 2 | 7 |
| 11.2 | Structural suggestions for improvement | 1 | 0 | 0 | 0 | 1 | 0 | 0 | 0 | 1 | 0 | 0 | 0 | 0 | 5 | 8 |
| **B. The nine main categories not selected for this sub-analysis (no inductive subcategories were developed within them)** | | | | | | | | | | | | | | | | |
| **12** | **Influences of Cultural, Social and Political Environment** | **10** | **1** | **1** | **19** | **9** | **4** | **4** | **5** | **1** | **6** | **1** | **0** | **4** | **1** | **66** |
| **13** | **Intrapersonal Changes after Psychedelic Treatment** | **8** | **1** | **2** | **11** | **10** | **3** | **3** | **4** | **6** | **0** | **0** | **2** | **1** | **8** | **59** |
| **14** | **Goals of Psychedelic Treatment** | **6** | **2** | **5** | **6** | **4** | **5** | **8** | **4** | **3** | **1** | **3** | **2** | **3** | **4** | **56** |
| **15** | **Background Knowledge** | **10** | **0** | **3** | **6** | **5** | **3** | **4** | **3** | **3** | **0** | **1** | **1** | **1** | **3** | **43** |
| **16** | **Social, Ethical and Political Concerns of Psychedelic Treatment** | **2** | **1** | **0** | **0** | **0** | **0** | **2** | **1** | **1** | **2** | **0** | **0** | **2** | **1** | **12** |
| **17** | **Interpersonal Changes after Psychedelic Treatment** | **3** | **0** | **2** | **1** | **0** | **1** | **0** | **0** | **0** | **0** | **0** | **0** | **0** | **1** | **8** |
| **18** | **Social, Ethical or Political Changes after Psychedelic Treatment** | **1** | **1** | **0** | **3** | **0** | **0** | **1** | **0** | **0** | **0** | **0** | **0** | **0** | **0** | **6** |
| **19** | **Spiritual, Religious, Philosophical and Metaphysical Changes after Psychedelic Treatment** | **2** | **0** | **0** | **0** | **1** | **0** | **1** | **0** | **0** | **0** | **0** | **0** | **0** | **0** | **4** |
| **20** | **Spiritual, Religious, Philosophical and Metaphysical Concerns of Psychedelic Treatment** | **2** | **1** | **0** | **0** | **0** | **0** | **0** | **0** | **0** | **0** | **0** | **0** | **0** | **0** | **3** |

Part A: 871 codings in the eleven main categories and 408 in their 36 subcategories. Part B: 257 codings in the nine remaining main categories.
